# Giant Moray Eel (*Gymnothorax javanicus*), a Long-Living Apex Predator That Poses a Food Safety Risk in the Pacific

**DOI:** 10.3390/md23090341

**Published:** 2025-08-26

**Authors:** Emillie M. F. Passfield, Kirsty F. Smith, D. Tim Harwood, Joshua D. Fitzgerald, Phoebe A. Argyle, Jacob Thomson-Laing, J. Sam Murray

**Affiliations:** 1 Cawthron Institute, Private Bag 2, Nelson 7040, New Zealand; kirsty.smith@cawthron.org.nz (K.F.S.); tim.harwood@cawthron.org.nz (D.T.H.); joshua.fitzgerald@cawthron.org.nz (J.D.F.); jacob.thomson-laing@cawthron.org.nz (J.T.-L.); 2 New Zealand Food Safety Science and Research Centre, Massey University, Private Bag 11 222, Palmerston North 4442, New Zealand; 3 Ministry of Marine Resources, Moss Rd, Avarua P.O. Box 85, Rarotonga, Cook Islands; p.argyle@mmr.gov.ck

**Keywords:** ciguatera poisoning, ciguatoxin, bioaccumulation, aging, bioactive, mass spectrometry, toxicity, elements, heavy metals, nutrition

## Abstract

The giant moray eel (GME; *Gymnothorax javanicus*) is an important marine species that plays a key ecological role in reef systems and is a valued food source for indigenous communities. However, it is well-known that GMEs pose a food safety risk due to their ability to accumulate high levels of ciguatoxins (CTXs), the toxins known to cause ciguatera poisoning. This study assessed the age, CTX levels, elemental composition, and nutritional profile of seven GME specimens collected from Muri Lagoon, Rarotonga (Cook Islands), representing the most detailed compositional investigation on this species. Age was determined for the three largest specimens, with the oldest being 39 years old. All specimens contained ciguatoxins, with Type I (CTX4A derivatives), Type II (CTX3C derivatives), algal-ciguatoxins, and biotransformed metabolites being detected. There was a higher CTX content in the liver samples compared to flesh samples, with the longest–heaviest specimen containing the highest levels. The CTX1B level observed in flesh samples of all seven eel specimens exceeded the recommended safe guidance level proposed by the USFDA. A similar ciguatoxin profile was observed across flesh sections, with the belly flap or top loin containing the highest levels of CTXs in most specimens. No bioactive metabolites produced by co-occurring harmful microalgae, including regulated shellfish toxins, were detected. Elemental analysis determined the presence of 21 elements, including arsenic, low levels of mercury, and the volcanic elements rubidium and strontium. Nutritionally, the GMEs were shown to be a lean protein source; however, due to the ubiquitous bioaccumulation of CTXs, they pose a food safety risk to consumers.

## 1. Introduction

Coastal communities around the globe face food safety risks from a variety of microbiological and chemical sources, which are heightened for small island nations that primarily rely on the marine environment for sustenance and trade. One of these is ciguatera poisoning (CP), the most significant non-microbial marine food-borne illness, with estimates of up to 50,000 people impacted a year [1]. Epidemiological studies performed within the South Pacific suggest this number represents only 2–10% of all cases due to underreporting and misdiagnosis [1,2,3]. CP is endemic to all tropical and subtropical regions of the world and poses a significant challenge in the Pacific region, impacting both local communities and global populations due to the increased reliance on export markets [4,5]. CP is caused by the consumption of fish contaminated with ciguatoxins (CTXs), potent neurotoxins produced by species from the epibenthic dinoflagellate genus *Gambierdiscus* [6,7,8,9,10]. The prevalence of CP not only jeopardizes the health and well-being of Pacific Islanders but also poses socioeconomic challenges due to its impact on local food sources, trade, and an increased burden on the healthcare system [11]. Rarotonga, an island in the Cook Islands archipelago, is a known CP hotspot [12]. It has a large lagoon that provides a shallow habitat with high macroalgal cover, resulting in a diverse range of *Gambierdiscus* spp. [13] and ciguatoxic fish species present on the reef system, in turn, heavily impacting local communities [12].

**Figure 1:**
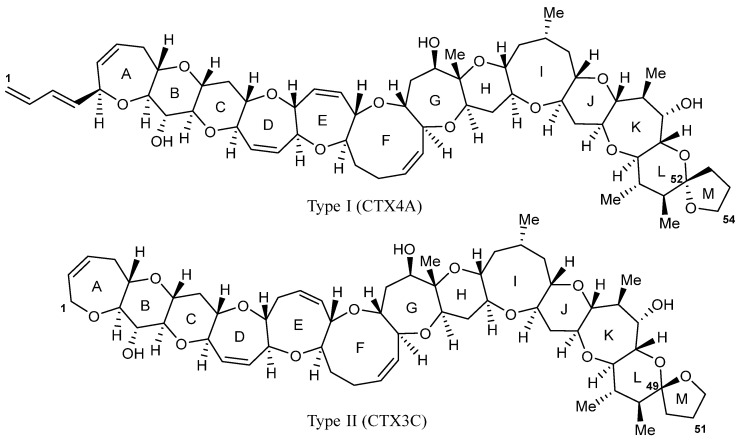
Example structures of CTX Type I (CTX4A), showing the aliphatic hydrocarbon chain on ring A, and Type II (CTX3C) with the eight-membered ring E.

In the Pacific region, CTXs are grouped into two distinct classes, namely, Type I (CTX4A derivatives) and Type II (CTX3C derivatives; Figure 1) [14]. Only two microalgal species (*G. polynesiensis* and *G. holmesii*) [14,15] have been chemically confirmed to produce Pacific CTXs; however, most *Gambierdiscus* species display some bioactivity when assessed using in vitro toxicity assays, indicating that there are additional toxins yet to be characterized [16]. Current dogma is that algal-CTXs enter the food web through herbivorous reef fish (e.g., parrotfish, *Chlorurus* spp.) that graze on substrates (e.g., corals, macroalgae) colonized by *Gambierdiscus* spp. The fat-soluble CTXs are subsequently bioaccumulated up the food chain when these fish are consumed by higher trophic levels, omnivorous and carnivorous species (e.g., giant moray eel, *Gymnothorax javanicus*) [17]. The algal-CTXs undergo a series of complex species-specific biochemical transformations, including oxidation, reduction, and conjugation, which increase their toxicity and, therefore, heighten the risk they pose to human health when consumed [18,19,20,21]. There is currently no internationally accepted ‘safe’ level of CTXs in seafood, with a guidance limit suggested by the United States Food and Drug Administration (USFDA) for the Pacific of 0.01 µg/kg CTX1B equivalence [22].

Studies have been conducted to investigate if there is a correlation between the toxicity, size, and age of fish, with the aim of enabling predictions to be made of the likelihood that a fish will cause illness in humans. Each study investigated different fish species from various locations, with some studies showing a positive correlation [23,24,25], while others demonstrated that there was no correlation between size and toxicity [26]. Where applicable in these studies, the fish were aged by counting the growth annuli of the otoliths [27], small calcium carbonate inner ear bones of bony fish, usually present in three pairs: asterisci, lapilli, and the largest pair, sagittae, which are most commonly used for aging. The calcified structures continue to form over the lifespan of the fish, where growth is increased in warmer periods and slowed in colder periods, consequently, producing tree-like growth rings [28].

Benthic microalgal communities within reef ecosystems are diverse, characterized by the coexistence of multiple genera and species. *Gambierdiscus* spp. are commonly found in assemblages on reef systems along with other benthic dinoflagellates, which produce a diverse range of potent bioactive metabolites that also represent potential food safety risks [29,30]. In the South Pacific, this includes some *Ostreopsis* spp. [31,32,33], which produce a range of structurally-related compounds known as palytoxin (PLTX)-like compounds, ostreocins (OST), and ovatoxins (OVTX) [34]; *Vulcanodinium* spp. that produce various cyclic imine compounds (e.g., pinnatoxins) that can bioaccumulate in marine species [31,32,33,35]; and *Prorocentrum* spp. that produce okadaic acid (OA) and dinophysistoxins (DTXs) [36,37,38]. The cohabitation of these microalgal genera, coupled with the production of a diverse range of bioactive metabolites, highlights the complexity and underscores the multifaceted risk that benthic harmful algae pose to food safety.

Another known food safety risk identified in some seafood species is heavy metals [39]. Lead, mercury, cadmium, and arsenic can be introduced into the environment through, for example, local volcanic activity or improper waste disposal [40,41]. Slow-growing, long-lived reef fish species that reside in an environment where heavy metals are present can accumulate levels of heavy metals that exceed health-based guidance values, and, therefore, pose a food safety risk if consumed regularly. Despite such risks, seafood represents a nutritious food source, and the risks of consuming it need to be balanced with its undoubted health-promoting attributes. Seafood is an essential food source for Pacific Island nations, high in protein, and a valuable commodity. Basic ‘proximate’ analysis, including moisture content, ash, protein, fat, and carbohydrates, provides baseline nutritional information about a particular species. Moisture content influences the shelf life and texture of the food; ash represents the total mineral content of the food, and total fat, protein, and carbohydrate determine the nutritional value and energy. All essential components when looking at a food source.

To better understand CP risk in Rarotonga (Cook Islands), a research expedition was undertaken in 2014 to perform a comprehensive benthic survey of the lagoon using a range of sampling techniques [42]. The biodiversity of *Gambierdiscus* species and bioaccumulation of Pacific CTXs in fish species from different trophic positions were mapped at six sampling locations around the island to elucidate the spatial extent of CP risk. This was achieved through sampling of various host macroalgal substrates, the deployment of artificial substrate samplers to capture potentially harmful algal species, and the collection of herbivorous, omnivorous, and carnivorous reef fish species. This expedition yielded significant findings, including the description of two new species of *Gambierdiscus*, *G. cheloniae* [10] and *G. honu* [43], the discovery of two new maitotoxin analogs, MTX-6 and MTX-7 [44], and four new gambierone analogs [14]. A variety of other *Gambierdiscus* species were isolated, all of which exhibited some level of in vivo toxicity [16]. Among these was *G. polynesiensis*, the only known species to produce the algal-CTXs in the Pacific region [9,14], which was found at all sampling sites, signaling its widespread presence and likely contribution to the local CP issue. Following on from the 2014 findings, a second expedition was undertaken in 2022 aimed at collecting similar benthic samples, including higher trophic level marine specimens, to evaluate changes in the risk of CP over time. During this expedition, seven giant moray eel (GME; *Gymnothorax javanicus*) specimens were collected, which are apex predators within reef ecosystems, occupy a unique ecological niche, and are a traditionally important food source to Pacific Island communities. However, GMEs have been implicated in severe CP illnesses in the South Pacific for decades [45,46] and are now rarely consumed in Rarotonga (per comms). CTX1B, the most potent CTX analog described to date, was first isolated and characterized from moray eels [47].

In this study, the seven specimens (GME 1–7) were comprehensively assessed for the presence of selected biogenic (CTXs) and geogenic (heavy metals) chemical contaminants that pose a food safety risk. The GMEs were assessed for the localized bioaccumulation profiles of the algal and biotransformed metabolites of the Type I and Type II CTXs, with the three largest aged using the sagitta otoliths. The presence of 62 other marine toxins produced by *Gambierdiscus* and common cohabitating harmful microalgae observed in Rarotonga was also investigated. Lastly, the bioaccumulation of heavy metals was determined, providing additional information on food safety risks, along with proximate determination to provide baseline nutritional information.

## 2. Results

### 2.1. Giant Moray Eel Identification

**Figure 2:**
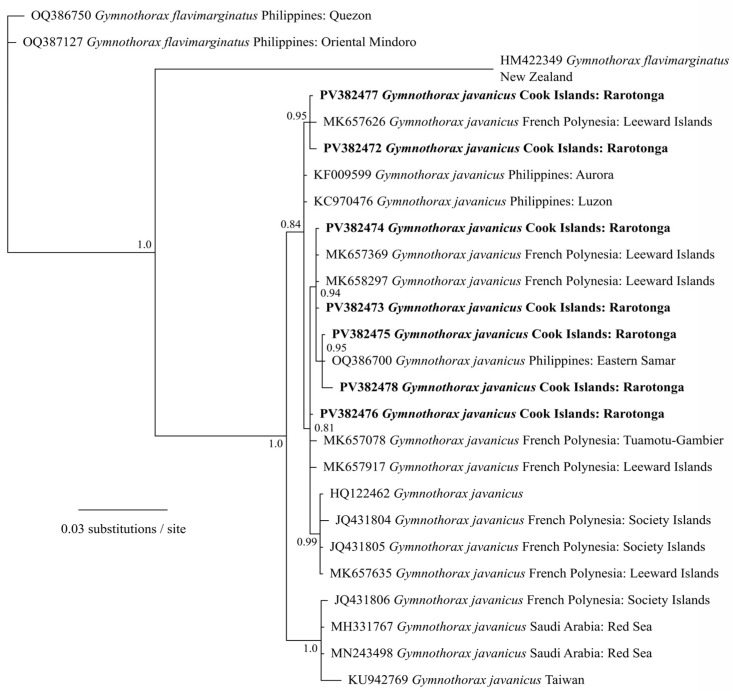
Phylogenetic analysis of mitochondrial cytochrome oxidase I (COI) gene sequences of the seven giant moray eels (*Gymnothorax javanicus)* specimens (in bold), collected from Muri Lagoon (Rarotonga, Cook Islands), analyzed with other *Gymnothorax* spp. sequences from GenBank using Bayesian analyses. Values at nodes represent Bayesian posterior probability support. Scale bar represents substitutions per site.

The specimens were first visually identified as GME based on the morphological characteristics. Three were tentatively identified as females based on the presence of roe, although no additional analysis was conducted to confirm this. Phylogenetic analysis of mitochondrial cytochrome oxidase I (COI) gene sequences confirmed that all seven GME specimens clustered with other *Gymnothorax javanicus* COI sequences from Asia and the Pacific with high support (Figure 2).

### 2.2. Aging

**Figure 3:**
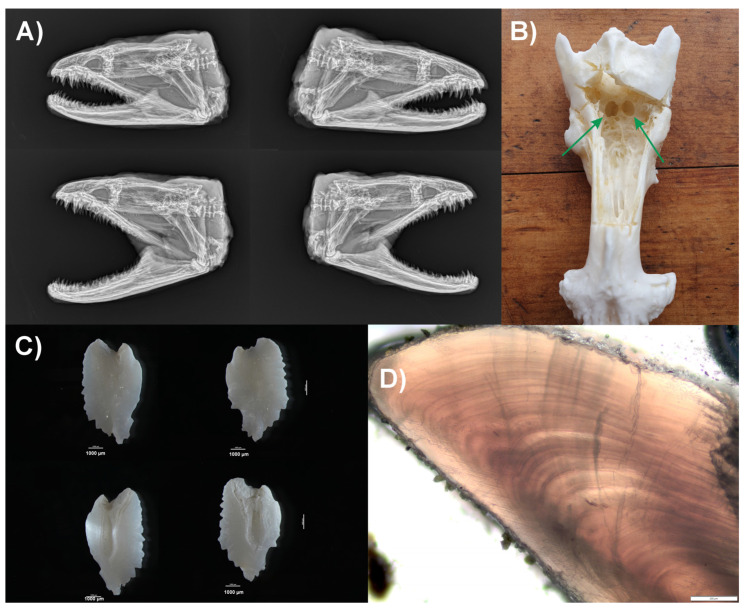
Images from the skull of the giant moray eel (*Gymnothorax javanicus*), specimen 2 (approximately 120 mm in length), collected from Muri Lagoon (Rarotonga, Cook Islands). (**A**) X-rays showing the left and right lateral views with the jaw closed and fully extended. (**B**) Photo of the neurocranium showing the location of the two sagitta otoliths (green arrows). (**C**) The left and right sagitta otoliths showing the concave dorsal depression and sulcal groove (convex side). (**D**) Cross-sectional view of the left-hand sagitta otolith used for aging.

The three largest GME specimens (GME 2, 3, and 7) were aged by counting the annuli of the sagitta otoliths. These were located using X-ray (GME 2 Figure 3A, GME 3 Figure S1, and GME 7 Figure S2), which showed that they were housed below the parietal bone in the posterior of the neurocranium (GME 2 Figure 3B). The sagitta otoliths were extracted (GME 2 Figure 3C, GME 3 Figure S3, and GME 7 Figure S4), and annuli were counted. GME 2 was determined to be 39 years old (Figure 3D), GME 3 was 26 years old (Figure S5), and GME 7 was 23 years old (Figure S6).

### 2.3. Sample Analysis for Ciguatoxins

#### 2.3.1. Screening

Flesh (left-hand fillet) and liver from the seven GME specimens were analyzed for 20 CTXs using an in-house liquid chromatography–tandem mass spectrometry (LC-MS/MS) method [48]. Reference material was available for twelve analogs (purified standards or CTX-positive fish and microalgal extracts). In the flesh, eight analogs were detected—CTX1B, 52-*epi*-54-deoxyCTX1B, 54-deoxyCTX1B, 2,3-dihydroxyCTX3B, 2,3-dihydroxyCTX3C, CTX3B (49-*epi*CTX3C), CTX3C, and M-*seco*-CTX3B/3C (Table 1). Of those detected, CTX3B was the most abundant metabolite in all GME specimens, ranging from 0.04–0.53 µg/kg, whereas CTX3C was identified in all but one specimen, ranging from 0.04–0.24 µg/kg. Notably, both Type I and Type II algal CTXs and biotransformed metabolites were detected in the flesh samples (Table 1). 2,3-dihydroxyCTX3B and 2,3-dihydroxyCTX3C were also identified in all seven GME specimens, with quantifiable levels (i.e., above the limit of quantitation [LoQ]) observed in five (0.04–0.34 µg/kg) and four (0.04–0.22 µg/kg) specimens, respectively. CTX1B was also observed in all seven GME specimens, but was only quantifiable in three, with concentrations ranging from 0.04 to 0.07 µg/kg. Of the eels examined, GME 2 had the highest concentration of CTX3B (0.53 µg/kg), CTX3C (0.24 µg/kg), 2,3-dihydroxyCTX3B (0.34 µg/kg), 2,3-dihydroxyCTX3C (0.22 µg/kg), and CTX1B (0.07 µg/kg). In addition, 52-*epi*-54-deoxyCTX1B, 54-deoxyCTX1B, and M-*seco*-CTX3B/3C were detected above the limit of detection (LoD) but below the LoQ in six, four, and five GME specimens, respectively. The algal metabolites CTX4A, CTX4B, and M-*seco*-CTX4A/B were not detected.

**Figure 4:**
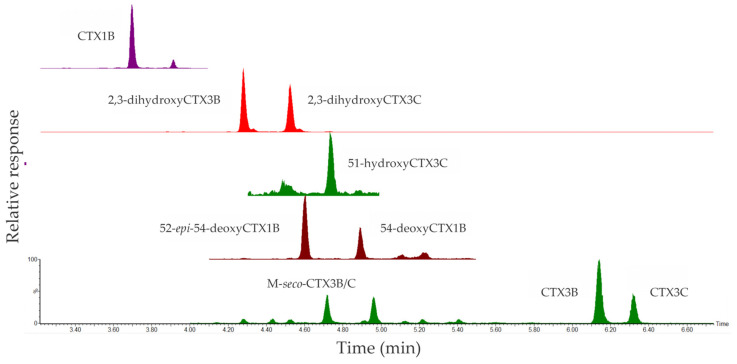
Stacked total ion chromatogram of ciguatoxins observed in the liver from the giant moray eel (*Gymnothorax javanicus*) specimen 2, collected from Muri Lagoon (Rarotonga, Cook Islands).

In the liver, nine CTX analogs were observed—CTX1B, 52-*epi*-54-deoxyCTX1B, 54-deoxyCTX1B, 51-hydroxyCTX3C, 2,3-dihydroxyCTX3B, 2,3-dihydroxyCTX3C, CTX3B, CTX3C, and M-*seco*-CTX3B/C (Figure 4). Of note was the presence of both Type I and Type II algal CTXs and biotransformed metabolites (Table 1). These were quantifiable in all GME specimens, with the only exception being that 51-hydroxyCTX3C was detected above the LoD but below the LoR in GME 1 and not detected in GME 6. The epimer pair 2,3-dihydroxyCTX3B and 2,3-dihydroxyCTX3C were the most abundant analogs, ranging from 6.0 to 54 µg/kg and from 8.4 to 55 µg/kg, respectively, with the highest concentration found in GME 2 (54 and 55 µg/kg, respectively). Trace levels of 51-hydroxyCTX3C were found in five GME specimens, ranging from 0.07 to 0.32 µg/kg, with the highest concentration found in GME 3. Algal metabolites CTX3B and CTX3C were also detected, ranging from 0.38 to 3.2 µg/kg and from 0.16 to 1.6 µg/kg, respectively. GME 2 contained the highest level at 3.2 and 1.6 µg/kg, respectively. M-*seco*-CTX3B/C was also present, ranging from 0.04 to 0.25 µg/kg, with GME 7 having the highest concentration. CTX1B concentrations ranged from 0.50 to 3.9 µg/kg, with the highest concentration found in GME 7. Comparatively, the concentrations of 52-*epi*-54-deoxyCTX1B and 54-deoxyCTX1B were much lower and ranged from 0.09 to 0.50 µg/kg and from 0.07 to 0.30 µg/kg, respectively. The highest concentrations were observed in GME 1 and GME 2, respectively. The algal metabolites CTX4A, CTX4B, and M-*seco*-CTX4A/B were not detected.

A sum of all CTX analogs quantified, referred to as ‘total CTXs’, ranged in the flesh from 0.04 µg/kg in GME 6 to 1.4 µg/kg found in GME 2. In the livers, the levels were approximately 100 times higher, ranging from 16 µg/kg in GME 6 to 118 µg/kg in GME 2. However, this is likely an underestimation as no recovery factors have been applied.

Eight additional CTX metabolites that have been reported in the literature were qualitatively assessed in the GME flesh and liver samples, with six tentatively identified based on published MRM transitions and relative retention times (Table 2; Table S2) [14,20,49]. Five of the analogs were found in all seven GME livers: 2,3,51-trihydroxyCTX3B/C, 7-oxo-CTX1B, 7-hydroxy-CTX1B, M-*seco*-CTX3B/C methyl acetate, and 4-hydroxy-7-oxo-CTX1B, with 2-hydroxyCTX3C being observed in five livers. M-*seco*-CTX3B/C methyl acetate was also detected in the flesh of six GME specimens.

Comparing the total CTX content with the age of the three GME specimens demonstrated that GME 2 was the oldest of the three (39 years old) and had the highest concentrations of CTXs in the flesh (1.5 µg/kg) and liver (118 µg/kg). However, while GME 3 was older than GME 7 (26 vs. 23 years old), it had a lower total CTX content in the flesh (0.44 vs. 0.65 µg/kg) and liver (46 vs. 66 µg/kg), respectively.

#### 2.3.2. Localized Bioaccumulation Investigation

**Figure 5:**
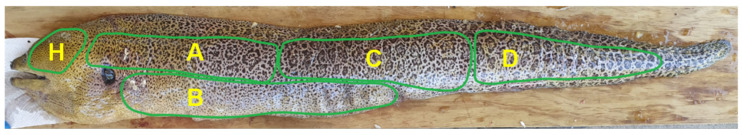
Flesh sections were analyzed to determine if there was localized ciguatoxin bioaccumulation in giant moray eel (*Gymnothorax javanicus*) specimens, collected from Muri Lagoon (Rarotonga, Cook Islands). (A) top loin, (B) belly flap, (C) center loin, (D) tail section, and (H) head flap for GME specimens 2, 3, and 7 only.

The right-hand fillet (looking head to tail) of all seven eel specimens was separated into and analyzed as four sections (Figure 5, sections A–D) to determine whether there was any CTX bioaccumulation. The head flap (Figure 5, section H) was also included for analysis for GME 2, GME 3, and GME 7.

**Figure 6:**
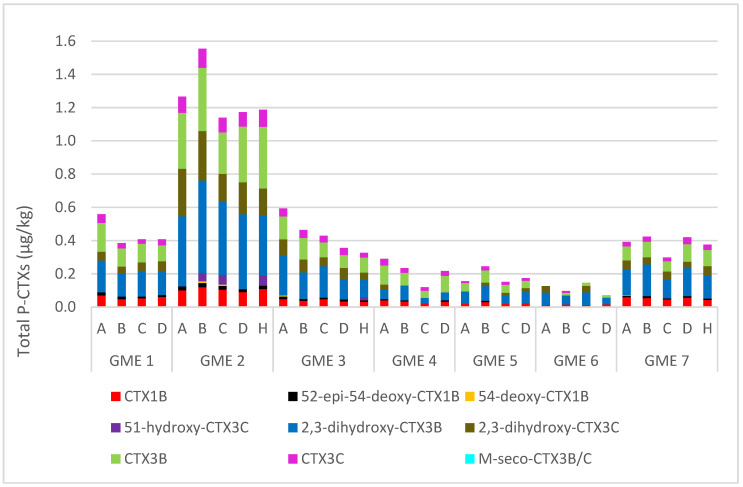
Total ciguatoxin content for the flesh sections in the seven giant moray eel (*Gymnothorax javanicus*) specimens, collected from Muri Lagoon (Rarotonga, Cook Islands). A = top loin, B = belly flap, C = center loin, D = tail section, and H = head flap.

Between three and nine CTX analogs were detected in the fillet sections A–D and head flaps of the GME specimens. The total CTXs, a sum of the CTXs quantified, were used to compare the five sections (Figure 6; Table S1). A similar CTX profile was observed across the different sections, with the top loin (section A) or belly flap (section B) having the highest CTX levels in GME 1 (0.54 µg/kg), GME 2 (1.52 µg/kg), GME 3 (0.57 µg/kg), GME 4 (0.28 µg/kg), and GME 5 (0.21 µg/kg). For GME 6, the highest CTX levels were observed in the top loin and center loin (section C; 0.11 µg/kg), and for GME 7, in the belly flap and tail section (section D; 0.41 µg/kg).

**Figure 7:**
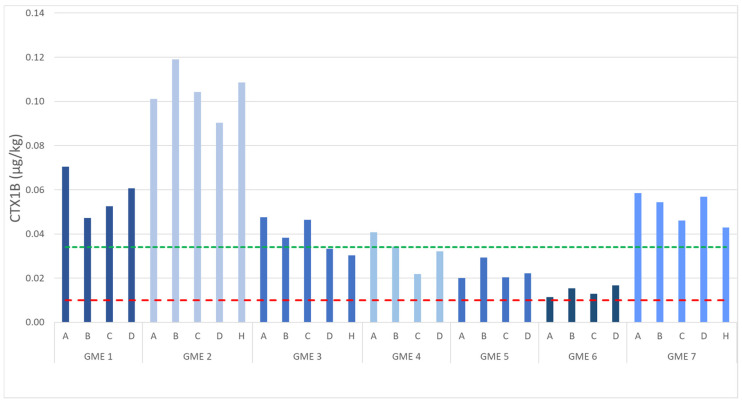
Comparison of ciguatoxin (CTX)1B levels observed in the flesh from the seven giant moray eel (*Gymnothorax javanicus*) specimens collected from Muri Lagoon (Rarotonga, Cook Islands). A = top loin, B = belly flap, C = center loin, D = tail section, and H = head flap. The green line represents the limit of quantitation, with values below this line reported as detected but not accurately quantified. The red line indicates the United States Food and Drug Administration-recommended guidance limit of 0.01 µg/kg CTX1B [22].

A similar trend was observed when comparing the concentration of the individual analogs, for example, CTX1B (Figure 7), with the top loin or belly flap (section B) having the highest CTX level in GME 1 (0.07 µg/kg), GME 2 (0.12 µg/kg), GME 3 (0.05 µg/kg), GME 4 (0.04 µg/kg), GME 5, GME 6 (highest relative detection; above the LoD but below the LoQ), and GME 7 (0.06 µg/kg). While these values are all at or above the USFDA guidance limit, they are an underestimation of the actual CTX quantity due to low recovery from the GME matrix and no recovery factors being applied (refer to Section 2.3.3).

#### 2.3.3. Fortification Experiments to Determine Method Performance

The analysis of CTXs in seafood samples is very challenging due to the low (parts per billion) levels typically observed, extreme lipophilicity, and difficulty removing co-extractives. For these reasons, it was important to determine the recovery of CTXs from the GME. Selected GME specimens were fortified with 1 µg/kg of four CTXs, representing Type I and Type II algal CTXs and biotransformed metabolites (CTX1B, CTX3B, CTX3C, and CTX4A). The fortification level was relatively high due to endogenous CTXs present in specimens. Fortification occurred pre-extraction to assess extraction efficiency/recovery (full fortification; Table 3), and a separate experiment fortified the sample extract post-SPE to assess matrix suppression and enhancement effects (post-SPE fortification; Table 3). Recoveries were calculated by subtracting the endogenous CTX content from the fortified samples and dividing by the fortified concentration.

Recovery of CTX1B was 88% for the fortified solvent (60% aq. MeOH), 68% for GME 1 flesh, 54% for GME 4 flesh, and 69% for GME 2 liver. The recovery of the more lipophilic algal metabolites CTX3B, CTX3C, and CTX4A for the fortified solvent ranged from 87 to 90%; however, it was much lower for the GME specimens, ranging from 19 to 28% in the flesh and from 9 to 49% in the liver. The poor recoveries for these analogs from the GME were expected as the extraction procedure was developed for the more hydrophilic biotransformed metabolites (e.g., CTX1B). Post-SPE fortification was performed on the flesh from three GME specimens, showing some matrix suppression with recoveries ranging from 74 to 97%. A post-SPE fortification of a liver sample showed heavy suppression for CTX1B, with a recovery of 44%, although the algal CTXs were relatively unaffected, with recoveries ranging from 79 to 94%. It is acknowledged that there are inherent caveats when spiking naturally incurred samples, such as interference from endogenous levels of CTXs and unknown binding affinities of the matrix compared to naturally bioaccumulated material.

### 2.4. Additional Marine Toxins

An additional 62 hydrophilic and lipophilic bioactive secondary metabolites were monitored in the GME specimens. This consisted of azaspiracids, brevetoxins, cyclic imines (spirolides, pinnatoxins, gymnodimine), diarrhetic shellfish toxins, domoic acid, gambierones, gambieric acids, gambierol, gambieroxide, maitotoxins, pectenotoxins, palytoxin-like compounds, and yessotoxins. There were no detections.

### 2.5. Elemental Composition in the Giant Moray Eel Flesh

The elemental composition of the GME was determined by analyzing the specimens for 34 elements, with 21 detected (Table 4). The five most abundant elements were potassium, phosphorus, sulphur, calcium, and sodium (in descending sequential order). Zinc, iron, and arsenic were present and ranged from 15 to 21 mg/kg, from 2.6 to 7.6 mg/kg, and from 1.6 to 4.9 mg/kg, respectively. Alkaline earth and alkali metals strontium and rubidium were also detected, with quantities ranging from 3.0 to 8.9 mg/kg and from 0.64 to 0.78 mg/kg, respectively. Additional elements detected were barium, chromium, caesium, copper, mercury, lithium, manganese, molybdenum, nickel, and selenium, with detections ranging from 0.012 to 1.0 mg/kg.

### 2.6. Proximate Composition in the Giant Moray Eel Flesh

The nutritional composition of GME flesh was determined for all seven specimens (Table 5). Fat content ranged from 1.8 to 6.6 g/100 g, protein from 18.1 to 20.4 g/100 g, moisture from 74.5 to 77.5 g/100 g, and ash from 1.1 to 1.7 g/100 g.

## 3. Discussion

This study investigated the seafood safety risk posed by GME using seven specimens collected using a hand speargun in Muri Lagoon, Rarotonga (Cook Islands). The GME were tentatively identified based on morphological characteristics, with phylogenetic analyses showing that COI sequences clustered with other *G. javanicus* specimens from Asia and the Pacific. This body of research focused particularly on naturally occurring chemical contaminants that pose a risk to human health, including both qualitative and quantitative LC-MS/MS analyses for bioactive metabolites produced by toxin-producing microalgal species and ICP-MS analyses for 34 elements, including several heavy metals. This represents the most detailed compositional analysis performed on this species to date.

Isolates of the dinoflagellate *G. polynesiensis* found in the Pacific region produce both Type I and Type II CTXs [14], which are biotransformed into more potent metabolites as they are bioaccumulated in the food web [20,21]. The GMEs were analyzed for CTXs and, in this study, revealed that all seven specimens had Type I, Type II, algal CTX, and biotransformed metabolites in their liver and flesh, which represents the first time such a concoction of CTXs has been reported in a seafood species. Of the metabolites observed, the Type II algal-CTX, CTX3B, and the biotransformed analogs, 2,3-dihydroxy-CTX3B and 2,3-dihydroxy-CTX3C, were the most abundant and detected in quantifiable levels. Interestingly, the Type I metabolite most frequently implicated in CP events, CTX1B, was only detected at lower concentrations and was only quantifiable in the flesh of three specimens. The poor recovery of the CTXs observed in the fortification experiments means that these results are underestimated and will, in fact, be higher in the GME specimens. The metabolites that typically co-occur with CTX1B, namely, 52-*epi*-54-deoxyCTX1B and 54-deoxyCTX1B, were only identified in six and four GME specimens, respectively. The USFDA has recommended a food safety guidance limit of 0.01 µg/kg CTX1B equivalence [22], but not for any of the other CTX analogs found in the Pacific to date. When coupled with the fact that no official toxicity equivalence factors exist for the other CTX analogs, it is not possible to convert the CTX levels observed into CTX1B equivalents. Therefore, comparing the concentration of CTX1B alone in the seven specimens is of most relevance, which showed that all flesh sections exceeded the USFDA guidance level, demonstrating that they are not suitable for consumption. However, this guidance level has been challenged by researchers in Japan who have determined a CTX limit in fish that is 17.5 times higher (0.175 µg/kg CTX1B equivalence) [50] than that proposed by the USFDA (0.01 µg/kg CTX1B equivalence) [22]. This highlights that additional research needs to be conducted, ideally with meal remnants, to determine an accurate and consistent CTX health-based guidance value. In addition, these limits only reference CTX1B; therefore, more toxicological information on the other CTX analogs is needed to accurately determine the risk they pose to seafood consumers.

To the best of our knowledge, there are no standardized methods for aging GME, although a common approach for other eel and fish species is counting otolith annuli (growth rings) [51,52,53]. Otolith analyses of the three largest GME specimens revealed that GME 2 was 39 years old, which also had the highest number of CTX analogs and the highest total CTX concentration found in the flesh and liver, and was the heaviest and the longest of the seven GME collected. GME 3 was 26 years old, and GME 7 was 23 years old, and although GME 7 was 3 years younger, this specimen had 50% more total CTXs in the liver and flesh compared to GME 3. In addition, GME 1 had the second-highest total CTX concentration in the flesh and liver, yet it was the second-smallest eel assessed. While these results are inconclusive to determine if there is a relationship between age and CTX content, they provide valuable insight into the age of GME found in Muri Lagoon, Rarotonga (Cook Islands), supporting their perceived status as a long-living apex predator within marine ecosystems [54,55].

Following initial analyses to confirm the GME contained CTXs, comparison of bioaccumulation in different flesh portions was performed. This involved one of the fillets being cut into four sections, with the head flap also included for the three largest specimens (GME 2, GME 3, and GME 7). There were between three and nine different CTXs analogs identified in the different sections of GME, with similar CTX profiles observed within the same GME and different between GME specimens. In most cases, the top loin or belly flap had the highest concentrations of total CTXs and the highest number of CTX analogs present. For individual CTX analogs, a similar pattern was observed: the top loin or belly flap sections showed the highest level of bioaccumulation. It is hypothesized that this is due to the higher concentration of lipids found in these sections, thereby having a greater affinity to the lipophilic CTX metabolites. However, the lipid content of the individual sections was not assessed as part of this study, so this still needs to be experimentally proven.

While the primary focus of this study was on the detection of CTXs in the GME specimens, analysis also targeted known hydrophilic bioactive metabolites produced by *Gambierdiscus* spp. found in Rarotonga, namely, maitotoxins, gambierones, gambieric acids, gambieroxide, and gambierol [14]. There were no detections of these compounds, suggesting that in the studied region, CTXs may be the predominant toxins present in the GME. However, the extraction procedure used was not developed for these hydrophilic metabolites, nor did the LC-MS/MS system have the resolution required to identify any novel biotransformed metabolites in the extracts. Therefore, it is currently unknown if the metabolites were not extracted from the matrix, if they are biotransformed into novel analogs unable to be detected on the LC-MS/MS system used, or if they simply do not bioaccumulate up the food chain due to their hydrophilic nature. Further research is warranted in this area.

The accumulation of bioactive metabolites produced by other common cohabitating microalgae was also investigated, which used extraction procedures developed in-house for the specific toxin classes and included azaspiracids, brevetoxins, cyclic imines, domoic acid, okadaic acids, pectenotoxins, palytoxin-like compounds, and yessotoxins. There were no detections of these additional metabolites, although it is important to consider the role of cohabitating species in the broader context of food safety risks. Various reef-associated species, including herbivorous fish and smaller predators, can harbor different microalgal toxins, contributing to a tapestry of toxicity in the marine ecosystem. These species could serve as vectors for other toxins, which may not have been detected in this study but could still pose risks to humans if they bioaccumulate in large fish predators like GME. The absence of other toxins in this small number of GME from a single location does not exclude the possibility of their presence in tropical reef ecosystems, particularly in species lower in the food chain.

In addition to toxins, there are many naturally occurring food safety risks from consuming marine fish, which are associated with different elements, including heavy metals. Of the 34 elements analyzed in this study, 21 were detected in the seven GME, indicating a complex composition likely influenced by local geological factors. Of the elements detected in the GMEs, there were high levels of total arsenic found in the flesh, with the highest levels observed in GME5, although these levels did not exceed the health-based guidance values for arsenic in fish [56]. Conversely, only low levels of mercury were detected in the GMEs, indicating that the GMEs primarily prey on lower trophic level species, rather than higher trophic level pelagic predators (e.g., sharks [57]) that are known to have elevated mercury concentrations. Volcanic emissions and weathering of volcanic deposits can contribute to the elements found in the marine environment [58,59], with the detection of alkaline earth and alkali metals in GMEs, such as strontium and rubidium, respectively, along with other trace elements, including barium, chromium, caesium, copper, lithium, manganese, molybdenum, nickel, and selenium [59], likely a result of the volcanic activity in the region.

The nutritional profile of GMEs is characterized by low fat and high protein and moisture contents, with no carbohydrates present, as determined by calculation. From a nutritional perspective, GME is a nutritionally sound food source for Pacific Islanders and one that has traditionally been a valued food source (per comms). However, due to the ubiquitous bioaccumulation of chemical contaminants and reported illnesses from consuming GME, the food safety risk posed by this species is substantial, leading to warnings not to consume GME in various Pacific Islands. Collectively, this highlights the importance of assessing the food value of GME but also for evaluating its role in human diets and potential food safety risks, especially in regions where seafood is heavily relied on as a staple protein source.

Further research to investigate if these observations are similar for other higher trophic level residential predators is now required, which will provide valuable information on the CTX bioaccumulation across the higher trophic levels of the marine food web.

## 4. Materials and Methods

### 4.1. Chemicals and Reagents

High-purity chromatography-grade methanol (MeOH), ethanol (EtOH), acetonitrile (MeCN), HPLC-grade dichloromethane (DCM), glacial acetic acid, and nitric acid (HNO_3_) were sourced from Thermo-Fisher Scientific (Fisher-Optima; Massachusetts, USA); analytical grade ammonia (≥25%), periodic acid, sodium hydroxide, and formic acid 98–100% are from Merck Life Science (Suprapur, Darmstadt, Germany), and purified water (18.2 MΩ) was produced in-house with a Milli-Q System (Millipore, Ontario, Canada). Hydrochloric acid (HCl) and high-purity *n*-hexane were purchased from Lab Supply Ltd. (Supelco, Auckland, New Zealand).

### 4.2. Giant Morey Eel Specimens

#### 4.2.1. Collection and Preparation

Seven GME specimens were collected using freediving and handheld spearfishing equipment from Muri Lagoon, Rarotonga (Cook Islands). They were photographed, measured, and dissected, with the livers and left and right fillets retained for each eel (Table 6 and Figure S7). The head flap and skulls were also retained from the three largest eels—GME 2, GME 3, and GME 7. The left-hand fillets from each GME were homogenized in their entirety using an industrial-grade mincer, while the right-hand fillets were portioned into four sections (top loin, belly flap, center loin, and tail section), and along with the head, flaps were homogenized individually. The livers were homogenized using a Breville stick blender (Breville, Sydney, Australia).

#### 4.2.2. Species Confirmation

A portion of the homogenized left-hand fillet from each GME was used for phylogenetic analysis and species confirmation. Tissue samples from each GME were extracted using Zymo Quick-DNA Miniprep extraction kits following the animal tissue protocol (Zymo Research, CA, USA). An approximately 650 bp section of the mitochondrial cytochrome oxidase I (COI) gene was amplified from the DNA extracts using the primers FishF1 (5′-TCAACCAACCACAAAGACATTGGCAC-3′) and FishR2 (5′-ACTTCAGGGTGACCGAAGAATCAGAA-3′) [60]. The PCR amplifications were carried out in 50 μL reaction volumes containing MyTaq™ 2x PCR master mix (Bioline, MA, USA), both forward and reverse primers (0.4 μM final concentration), and template DNA (ca. 50–150 ng). Thermocycling conditions were an initial step of 2 min at 95 °C, followed by 35 cycles of 30 sec at 94 °C, 30 sec at 54 °C, and 1 min at 72 °C, followed, in turn, by 10 min at 72 °C. Sanger sequencing was carried out by an external contractor (Genetic Analysis Services, University of Otago, Dunedin). Sequences were assembled and aligned with other *Gymnothorax* spp. COI sequences from GenBank in Geneious Prime^®^ v2019.2.3 [61] using ClustalW 2.1 [62]. Bayesian analyses were carried out in Geneious using MrBayes 3.1.2 [63] using the evolutionary model (general time reversible with gamma-distributed rate variation across sites and a proportion of invariable sites, GTR + I + G). Analyses of alignments were carried out in two simultaneous runs with four chains each, 2.1 × 10^6^ generations, sampling every 1000 trees. A 50% majority-rule consensus tree was drawn from the last 1000 trees. All final split frequencies were <0.01.

#### 4.2.3. Otolith Preparation and Age Determination

The age of the three largest eels (GME 2, GME 3, and GME 7) was determined by counting the annuli of the sagitta otoliths [27,28], which were located using X-ray imaging of the skulls at Cawthron’s Aquaculture Park, Nelson, New Zealand. An Atomscope HFX90V EX9025V portable X-ray unit (DLC Australia Pty, Ltd., Melbourne, Australia) coupled with a Canon CXDI-410C Wireless Cesium Amorphous Silicon digital radiographic receptor (DLC Australia Pty, Ltd., Melbourne, Australia; image area = 430 × 420 mm, resolution = 3408 × 3320 pixels, pixel pitch = 125 um) and positioned at 50 cm distance was used set at 60 kV and 0.1 mAs^−1^. The skull cap was carefully cut using a Dremel with a circular saw blade, and the otoliths, which are below the parietal bone in the posterior of the neurocranium, were removed, washed with Milli-Q water and ethanol, and dried at room temperature. Photographs were taken using a Nikon SMZ800 microscope coupled with a Nikon DS-Ri2 camera.

The otoliths were prepared using the cut-and-polish technique: The cleaned otoliths were embedded in Crystalbond 509 (a thermolabile mounting adhesive) on a transparent microscope slide, which was left to fully cure. Using an Olympus IX53 inverted light microscope, the nuclei of each otolith were located, and using a heated scalpel, the otolith was bisected (offset from the nuclei) and remounted on the sagittal plane with Crystalbond 509. The convex proximal face was polished using a series of progressively finer abrasive sandpapers starting at 50 µm. The Crystalbond 509 was reheated, the otolith flipped onto its flat, polished edge, and the concave distal face was polished using the same series of sandpapers. This occurred several times until final polishing was performed with a fine 3 µm metallurgical lapping film.

The polished otolith sections were examined using an inverted light microscope, and the annuli were counted by three analysts to determine the age of each GME. It was assumed that each colored ring represents one year of growth.

### 4.3. Toxin Analysis

#### 4.3.1. Extractions

Ciguatoxins: GME flesh and liver samples were extracted as per Murray et al. [48] to look for algal-CTX and biotransformed metabolites. In brief, 5 g ± 0.1 g subsamples of homogenized GME flesh or liver were extracted using 15 mL 60% aq. MeOH (3:2 *v*/*v*) and Ultra-Turrexed (IKA, Guangzhou, China) for 1 min at 15,000 rpm in an ice bath. The slurry was cooked at 100 °C in a water bath for 10 min, rapidly cooled in an ice bath, and centrifuged at 3200× *g* for 10 min at 4 °C. A 5 mL aliquot of the supernatant was transferred to a 15 mL graduated chemical-resistant polypropylene tube, and a liquid–liquid partition was performed with 5 mL DCM. The top aqueous MeOH layer and the first 1 mL of DCM were aspirated to waste, leaving 4 mL of DCM in the sample tubes. The samples were then taken to dry under a continuous stream of N_2_ at 50 °C to ensure no aqueous portion remained. The dried extract was redissolved in 4 mL of DCM, loaded onto a preconditioned (10 mL of DCM) aminopropyl SPE cartridge (CUNAX123; 200 mg/3 mL; from United Chemical Technologies, Levittown, NY, USA), washed with 4 mL of DCM, and eluted with 4 mL of DCM:MeOH (9:1 *v*/*v*) into a fresh 15 mL graduated polypropylene tube. All SPE steps were performed without the need for vacuum pressure. The eluted extracts were taken to dry under a continuous stream of N_2_ at 50 °C, redissolved in 200 μL of 80% aq. methanol (4:1 *v*/*v*), and transferred into an autosampler glass insert ready for LC-MS/MS analysis.

Palytoxin-like compounds, GME flesh (GME 2, GME 3, and GME 7), and liver (GME 2) were extracted using an in-house developed method for intact palytoxin (PLTX), ovatoxin (OVTX), and ostreocins (OST; manuscript in preparation). In brief, 2 g ± 0.1 g subsamples of either the homogenized left-hand fillet or liver were extracted using 18 mL of 50% aq. MeOH (1:1 *v*/*v*), blended using an Ultra-Turrex (IKA, Guangzhou, China) at 15,000 rpm for 1 min in an ice bath and clarified using centrifugation at 3200× *g* for 10 min. A 6 mL aliquot of the supernatant was loaded onto a preconditioned (3 mL MeOH followed by 3 mL Milli-Q water) Strata-X 33 µm Polymeric Reversed Phase SPE cartridge (60 mg/3 mL; from Phenomenex, Torrance, CA, USA), washed with 3 mL 40% aq. MeOH (2:3 *v*/*v*) and eluted with 3 mL 80% aq. MeOH (4:1 *v*/*v*) containing 0.1% acetic acid. An aliquot of the eluent was transferred to an autosampler vial ready for LC-MS/MS analysis.

In addition to intact PLTX-like extraction, the GME specimens were also extracted and prepared using a modified version of the oxidative cleavage method described in Selwood et al. [64]. Using the 50% aq. The MeOH extract generated above, a 6 mL aliquot of the supernatant, was loaded onto a preconditioned (3 mL MeOH followed by 3 mL Milli-Q water) Strata-X 33 µm Polymeric Reversed Phase SPE cartridge (60 mg/3 mL; from Phenomenex, California, USA) and washed with 3 mL 40% aq. MeOH (2:3 *v*/*v*) and 3 mL Milli-Q water prior to on-column oxidation with 2 mL 50 mM periodic acid. The SPE cartridges were then washed with 2 mL Milli-Q water and eluted with 3 mL 60% aq. MeOH (3:2 *v*/*v*) containing 0.1% acetic acid. An aliquot of the eluent was transferred to an autosampler vial ready for LC-MS/MS analysis.

Other toxin classes: GME flesh (GME 2, GME 3, and GME 7) and liver (GME 2) were extracted and analyzed for 27 lipophilic shellfish and algal toxins and their metabolites using McNabb et al. [65]. In brief, 2 g ± 0.1 g subsamples of either the homogenized left-hand fillet or liver were extracted using 18 mL of 90% aq. MeOH (9:1 *v*/*v*) blended using an Ultra-Turrex (IKA, Guangzhou, China) at 19,000 rpm for 1 min in an ice bath and clarified using centrifugation at 3200× *g* for 10 min. A 2 mL aliquot of the supernatant was transferred to a 15 mL glass Kimax tube and a liquid–liquid partition performed with 5 mL *n*-hexane. The samples were centrifuged at 2500× *g* for 10 min, and ~1 mL of the lower aq. The MeOH layer was transferred to an autosampler vial ready for LC-MS/MS analysis.

To determine the total quantity of the diarrhetic shellfish toxins (DSTs), okadaic acid (OA), dinophysistoxins (DTX) -1 and -2, the samples were hydrolyzed to cleave the esterified forms following the procedure in Mountfort et al. [66]. In brief, a 1 mL aliquot of the crude extract (prior to partitioning with *n-*hexane) was subjected to base hydrolysis with 125 µL 2.5 M sodium hydroxide, heated at 76 °C for 40 min, cooled in an ice bath, and ‘neutralized’ using 125 µL of 2.5 M acetic acid. The samples are then centrifuged at 17,000× *g* for 5 min and diluted 1:4 with 80% aq. MeOH (4:1 *v*/*v*) into autosampler vials ready for LC-MS/MS analysis.

#### 4.3.2. Chromatographic Conditions and Instrumental Analysis

Liquid chromatography mass spectrometry analyses were conducted using a Waters Xevo TQ-S triple quadrupole mass spectrometer connected to a Waters Acquity UPLC i-Class system (Waters, Milford, MA, USA) equipped with a flow-through needle sample manager. The mass spectrometer operated in both positive and negative ion modes using electrospray ionization (ESI).

Unless stipulated otherwise, chromatographic separation was carried out on a Waters Acquity UPLC BEH phenyl column (1.7 µm, 100 × 2.1 mm) maintained at 50 °C. The column was eluted with mobile phases consisting of 0.2% (*v*/*v*) of a 25% NH_4_OH solution in Milli-Q water (A) and 95% aq. MeCN (9.5/0.5 *v*/*v*) (B). Fresh mobile phases were prepared daily to ensure optimal sensitivity and stable retention times. The autosampler chamber was held at 10 °C, with an injection volume of 2 µL. Additional settings included a cone voltage ranging from 30 to 75 V, a capillary voltage of 3.0 kV, a source temperature of 150 °C, a nitrogen gas desolvation flow rate of 1000 L/h at 600 °C, a cone gas flow rate of 150 L/h, and an argon collision cell flow rate of 0.15 mL/min. Data acquisition was performed with MassLynx V4.1 and processed using TargetLynx V4.1 software, respectively.

Ciguatoxins: The initial solvent composition was set at 5% B, with a linear gradient increasing to 50% B from 1 to 3.5 min, then to 75% B from 3.5 to 7.5 min. It was ramped up to 95% B by 8 min and maintained at 95% B until 9 min, followed by a return to 5% B at 9.2 min. The column was re-equilibrated with 5% B for 0.8 min. The total run time for each injection was 10 min, with a flow rate of 0.55 mL/min.

Quantitation was performed using five-point linear regression calibration curves (R^2^ > 0.98) of a mixed CTX standard (CTX3B, CTX3C, CTX4A, and CTX1B; Institute of Louis Malarde; linear range of 0.5–10 ng/mL), which were forced through zero. Where standards were unavailable, the calibration curve of the most structurally similar CTX was used for quantitation, with a relative response factor of 1. Reference material (in-house ciguatoxic microalgal and fish extracts) was used to confirm the presence of M-seco-CTX3B/C, CTX4B, M-seco-CTX4A/B, 52-*epi*-54-deoxyCTX1B, 54-deoxyCTX1B, 51-hydroxy-CTX3C, 2,3-dihydroxy-CTX3B, and 2,3-dihydroxy-CTX3C, based on MRM transition ratios and retention time. The extracts were also qualitatively analyzed for additional CTX metabolites using MRM transitions reported in the literature [17,20]; however, without reference material, the retention time of the CTXs is based on the likely lipophilicity of the CTXs compared to that of the known ones, and any detections are tentative only. A list of the MRM transitions, ESI mode, cone voltage, collision energy, and dwell times used for the metabolites is shown in Table S2.

Gambierones: Initial solvent composition of 5% B, a linear gradient to 50% B from 1 to 2.5 min, ramped up to 95% B by 3 min, and held at 95% B until 3.2 min, followed by a linear gradient back to 5% B at 3.5 min. The column was then re-equilibrated with 5% B for 0.5 min. The total injection time was 4 min, with a flow rate of 0.55 mL/min.

Quantitative analysis was performed using a six-point linear regression calibration curve (R^2^ > 0.98) of an in-house qNMR 44-MG standard (linear range of 5–1000 ng/mL) that was forced through zero. An experimentally determined relative response factor of 1 was used to quantify the gambierone cell quotas. In-house purified gambierone material [67] was used to confirm the retention time and confirmation ratio between MRM transitions. The remaining gambierone analogs were qualitatively assessed (Table S3).

Maitotoxins and gambieroxide: The initial solvent composition was 5% B, with a linear gradient to 30% B from 0.5 to 1 min, a linear gradient to 50% B from 1 to 3 min, ramped up to 95% B by 3.5 min and held at 95% B until 3.7 min, followed by a linear gradient back to 5% B at 4 min. The column was then re-equilibrated with 5% B for 0.5 min. The total injection time was 4.5 min with a flow rate of 0.5 mL/min.

The MTXs were quantified using a six-point linear regression calibration curve (R^2^ > 0.98) of an MTX-1 standard (Fujifilm Wako Pure Chemical Corporation; 2.5–200 ng/mL) that was forced through zero. A relative response factor of 1 was used to quantify MTX-5, MTX-6, and MTX-7. In-house purified MTX-6 and MTX-7 materials were used to confirm the retention time and confirmation ratio between MRM transitions. Analysis of gambieroxide was qualitative, with purified material (gifted from Ryuichi Watanabe) used to confirm the retention time (Table S3).

Gambieric acids and gambierol: The initial solvent composition was 5% B, with a linear gradient to 95% B from 0.5 to 3.8 min, held at 95% B until 4.2 min, followed by a linear gradient back to 5% B at 4.5 min. The column was then re-equilibrated with 5% B for 0.5 min. The total injection time was 5 min.

Detection of gambieric acids A–D and gambierol was qualitative only as no standards were available (Table S3). A culture extract of *G. australes* CAWD381 was used to confirm the retention time and confirmation ratio between MRM transitions of the gambieric acids.

Palytoxins: Chromatographic separation was carried out on a Waters Acquity UPLC BEH C18 column (1.7 µm, 50 × 1 mm) maintained at 25 °C. The column was eluted with mobile phases containing 0.1% (*v*/*v*) acetic acid in Milli-Q water (A) and MeCN (B). Fresh mobile phases were prepared daily to ensure optimal sensitivity and stable retention times. The autosampler chamber was held at 10 °C, with an injection volume of 2 µL for the intact palytoxin-like compounds and 3 µL for the oxidative cleavage method. Additional settings included a cone voltage of 30 V, a capillary voltage of 1.0 kV, a source temperature of 150 °C, a nitrogen gas desolvation flow rate of 1000 L/h at 500 °C, a cone gas flow rate of 150 L/h, and an argon collision cell flow rate of 0.15 mL/min. Data acquisition was performed with MassLynx and processed using TargetLynx software, respectively.

The initial solvent composition for the intact method was set at 5% B, with a linear gradient increasing to 25% B from 1 to 6 min, then to 90% B from 6.2 min and held until 7 min before returning to 5% B at 7.2 min. The column was re-equilibrated with 5% B for 1.3 min. The total run time for each injection was 9.5 min, with a flow rate of 0.2 mL/min.

Quantitation was performed using five-point linear regression calibration curves (R^2^ > 0.98) of a mixed PLTX (Wako standard), OST-D, and OVTX-a standard (as gifts from collaborators) with a linear range of 2–50 ng/mL, which were forced through zero. Prior to LC-MS/MS analysis, the concentration of the OVTX-a standard was determined using the UV spectrophotometry method detailed in Moore et al. [68], whereby the molar extinction coefficient was used to calibrate it against the Wako PLTX standard. Where standards were not available, microalgal extracts were used to confirm MRM transition ratios and retention times [69,70], with an assumed relative response factor of 1. A list of the MRM transitions, ESI mode, cone voltage, collision energy, and dwell times used for the metabolites is shown in Table S4.

For the oxidative cleavage method [64], the column oven temperature was increased to 40 °C, and the flow rate was increased to 0.25 mL/min. The initial solvent composition was 0% B, held for 0.5 min, ramped to 60% B over 2 min, then increased to 100% B between 2.5 and 3 min, before returning to 0% B at 3.2 min and re-equilibrating for a total injection length of 4 min.

Oxidized working standards of PLTX, OST-D, and OVTX-a were prepared as per Selwood et al. [64] and had a linear range of 2–50 ng/mL. Quantitation was performed for both the common PLTX-like amine fragment and the specific amide fragments corresponding to different PLTXs, OSTs, and OVTXs, using five-point linear regression calibration curves (R^2^ > 0.98) that were forced through zero. Microalgal extracts were used for the confirmation of retention times of compound-specific amide fragments, where reference material was unavailable. A list of the MRM transitions, ESI mode, cone voltage, collision energy, and dwell times used for the metabolites is shown in Table S5.

Other toxin classes: Chromatographic separation was carried out on a Waters Acquity UPLC BEH Shield RP18 (1.7 µm, 50 × 2.1 mm) and maintained at 40 °C. The column was eluted with mobile phases containing 2 mM NH_4_OH and 50 mM formic acid in 5% aq. MeCN (0.5/9.5 *v*/*v*) (A) and 90% aq. MeCN (9/1 *v*/*v*) (B). The autosampler chamber was held at 10 °C, with injection volumes of 1 µL for the non-hydrolyzed compounds and 2 µL for the hydrolyzed extracts. Additional settings included a cone voltage ranging from 30 to 80 V, a capillary voltage of 1.0 kV, the ESI source temperature at 150 °C, a nitrogen gas desolvation flow rate of 1000 L/h at 600 °C, a cone gas flow rate of 150 L/h, and an argon collision cell flow rate of 0.15 mL/min. Data acquisition was performed using MassLynx, and data processing was conducted with TargetLynx software. A list of the MRM transitions, ESI mode, cone voltage, collision energy, and dwell times used for the metabolites is shown in Table S6.

The initial solvent composition for the non-hydrolyzed method was set at 0% B and maintained for 0.2 min, followed by an increase to 15% B at 0.5 min, 30% B at 1 min, 80% B at 5 min, and 100% B at 7.5 min. The column was returned to initial conditions at 8 min and re-equilibrated for 0.6 min. The total run time for each injection was 8.6 min, with a flow rate of 0.5 mL/min.

For the analysis of the hydrolyzed extracts, the initial solvent composition was 40% B and increased to 70% over 1.5 min. At 2.2 min, the composition had ramped to 90% B, was 100% B by 2.5 min, and held for 0.5 min before returning to initial conditions at 3.5 min and re-equilibrated for 0.7 min. The total run time for each injection was 4.2 min, with a flow rate of 0.5 mL/min.

Certified reference material purchased from the National Research Council of Canada, Halifax, Nova Scotia, Canada, for domoic Acid (DA), desmethyl Spirolide-C (SPX-1), gymnodimine (GYM), OA, pectectontoxin-2 (PTX-2), and yessotoxin (YTX) was used for quantitative analysis. Desoxy-Brevetoxin-B2 (desoxy-BTX-B2) was sourced from Cawthron Natural Compounds, Nelson, New Zealand. A mixed stock at 10 µg/mL (DA and YTX) and 1 µg/mL (the remaining analytes) was used to prepare 5-point linear regression calibration curves (R^2^ > 0.98) from 200/20 ng/mL to 2/0.2 ng/mL, respectively. Where certified reference material was unavailable, quantitation was achieved using relative response factors (RRF) and toxicity equivalency factors (TEF). Azaspiracids-1, -2, and -3, Pinnatoxins a-g, and spirolide-D were quantified off SPX-1, BTX-B2 was quantified off desoxy-BTX-B2, homo-YTX, 45-hydroxy-YTX, and 45-OH YTX were quantified off YTX, and PTX-1, -6, -11, and PTX2 seco acid forms were quantified off PTX-2. The quantitation of free and esterified forms of DTX-1 and DTX-2 was quantified off OA. A list of available certified standards and the associated RRFs and TEFs is detailed in Table S7.

### 4.4. Elemental Analysis

Elemental analysis of the GME was conducted by the Cawthron Institute Food Chemistry laboratory, Nelson, New Zealand. The extraction and analytical methods were developed in-house and performed under ISO17025 accreditation.

#### 4.4.1. Extraction

A 2 g ± 0.1 g subsample of the homogenized left-hand fillet was weighed into a 50 mL Digi-tube (SCP Science, Baie-d’Urfé, QC, Canada) followed by 2.5 mL nitric acid, and 0.5 mL hydrochloric acid was added to each Digi-tube, which was then heated at 85 °C for 60 min using a hot-block digestion system (SCP Science, Baie-d’Urfé, QC, Canada). The Digi-tubes were cooled, and Milli-Q water was added to bring the total volume to 50 mL. Trace elements are determined on 20 dilutions of the digest solution using inductively coupled plasma mass spectroscopy (ICP-MS).

#### 4.4.2. Instrumental Analysis

Inductively coupled plasma mass spectrometry analyses were conducted using a Perkin Elmer Nexion 2000 ICP-MS (Perkin Elmer Inc., Waltham, MA, USA). The instrument was configured with a Cetac ASX-560 auto-sampler (CETAC Technologies, Omaha, NE, USA), ESI FAST sample introduction valve, Peltier cooled PC3 spray-chamber, and sea spray nebulizer.

Standard mode was used to acquire Boron at mass ^10^B, Barium at mass ^137^Ba, Beryllium at mass ^9^Be, Bismuth at mass ^209^Bi, Calcium at mass ^43^Ca, Cadmium at mass ^111^Cd, Mercury at mass ^202^Hg, Lanthanum at mass ^139^La, Lithium at mass ^7^Li, Lutetium at mass ^175^Lu, Molybdenum at mass ^98^Mo, Rubidium at mass ^85^Rb, Antimony at mass ^121^Sb, Strontium at mass ^88^Sr, Thallium at mass ^205^Ti, Uranium at mass ^238^U, Yttrium at mass ^89^Y, and Lead at mass ^206^Pb by adding the equation + ^207^Pb + ^208^Pb to the ^206^Pb isotope result. This has the effect of averaging out any variances in isotopic abundances. The following internal standards were used: Iridium (for Antimony, Bismuth, Mercury, Thallium, Lead, Yttrium, Uranium), Rhodium (for Cadmium, Molybdenum, Strontium, Rubidium), Scandium (for Boron, Beryllium, Calcium, Lithium), and Thulium (for Barium, Caesium, Lanthanum).

Kinetic Energy Discrimination mode was used to acquire Arsenic at mass ^75^As, Cobalt at mass ^59^Co, Chromium at mass ^52^Cr, Copper at mass ^63^Cu, Iron at mass ^56^Fe, Potassium at mass ^39^K, Magnesium at mass ^24^Mg, Manganese at mass ^55^Mn, Sodium at mass ^23^Na, Nickel at mass ^60^Ni, Phosphorus at mass ^31^P, Sulphur at mass ^34^S, Selenium at mass ^78^Se, Vanadium at mass ^51^V, Zinc at mass ^66^Zn The following internal standards were used: Scandium (for Cobalt, Chromium, Copper, Iron, Potassium, Magnesium, Manganese, Sodium, Nickel, Phosphorus, Sulphur, Vanadium, Zinc) and Tellurium (for Arsenic and Selenium).

### 4.5. Proximates

Proximate analysis was conducted by the Cawthron Institute Food Chemistry laboratory, Nelson, New Zealand, following official AOAC methods and performed under ISO17025 accreditation, unless stated in the sections below.

#### 4.5.1. Crude Protein

The crude protein content of the GME was determined using a modified version of AOAC’s official method 981.10. In brief, 2 g ± 0.1 g subsamples of the homogenized left-hand fillet were weighed out on Whatman 2122 paper and transferred to a digestion tube with boiling chips, catalyst tablets, 15 mL concentrated sulfuric acid, and 10 mL 30% hydrogen peroxide. The digestion was completed on a preheated digestion rack at 410 °C for 45 min, followed by the addition of 50–75 mL reverse osmosis water. Following digestion, distillation was performed on a K-355 unit, with the distillate collected in an Erlenmeyer flask containing boric acid and indicator solution. The solution was then titrated with 0.5 N sulfuric acid, with the end of the reaction determined by a color change from blue to pink. The percentage of crude protein was then calculated utilising the following equation:

(1) $$\frac{\left(sample\;titre-blank\;tire\right)\times 1.4007\times M}{Sample\;weight\;(g)}=\%Crude\;Protein$$

where 4.377 = 1.4007 × 0.5 × 6.25 (N);

1.4007 = milliequivalent weight N × 100 (%);

M = molarity of standardized acid (0.5);

N = 6.25 (protein factor for meat products (16% N).

#### 4.5.2. Total Fat

The total fat content of the GME was determined using the AOAC official method 948.15. In brief, 8 ± 0.1 g subsamples of the homogenized left-hand fillet were hydrolyzed with 10 mL hydrochloric acid to free bound fat in cell membranes and placed on a steam bath for 20 min (a blank extraction was performed using 4.4 mL Milli-Q water and was used for the final calculation). Once cooled to ambient temperature, the total fat was extracted from the aqueous solution with 10 mL of ethanol, and the acid hydrolyzed solution was transferred to Roese–Gottlieb extraction tubes, rinsed with 2 × 15 mL aliquots of diethyl ether, which were added to the extraction tube. The tube was shaken for 1 min before performing a liquid–liquid partition with 25 mL petroleum ether. The top ether–fat phase was decanted into a clean vessel, and two further partitions with the addition of 30 mL diethyl ether were performed, with the ether–fat layer collected each time. The samples are evaporated to dryness in a 105 °C oven for 40 min, cooled in a desiccator for 30 min, and weighed. The total fat percentage is calculated using the equation below:

(2) $$\frac{(\left(Weight\;fat+flask\right)-(Weight\;flask))-blank\;residue)\;\times 100}{Weight\;of\;sample\;(g)}=\%\;total\;fat$$

#### 4.5.3. Moisture at 105 °C

The moisture content of the GME was determined using a modified version of AOAC’s official method 950.46. A 2–3 g subsample of the homogenized left-hand fillet was spread thinly in an aluminium tray and heated at 105 °C for a minimum of 16 h to evaporate the water, leaving a dry residue. The samples were transferred to a desiccator, cooled for 15 min, and weighed. The difference in weight is the water lost by the sample using the following equation:

(3) $$100-\frac{\left(\;Weight\;Residue\;\times 100\right)}{Weight\;sample\;(g)}=\%\;moisture$$

#### 4.5.4. Ash

The ash content of the GME was determined using the AOAC official method 923.03. In brief, 3–5 g of homogenized left-hand fillet was placed in a crucible and placed in a furnace overnight at 550 °C. The samples were transferred to a desiccator, cooled for 45 min, and weighed. To calculate the % ash in the sample, the following equation was used:

(4) $$\frac{\left(Weight\;crucible+ash\right)-weight\;crucible\;}{Sample\;Weight\;(g)}\times 100=\%\;ash$$

## 5. Conclusions

This study represents the most comprehensive characterization performed on the giant moray eel (GME; *Gymnothorax javanicus*), shedding light on the longevity, food safety, and nutritional attributes of this apex predator. Seven GME specimens were collected from Muri Lagoon, Rarotonga (Cook Islands), which ranged in size and age, with the oldest being 39 years of age and 165 cm long. LC-MS/MS analysis revealed there were eight Type I and Type II algal CTXs and biotransformed metabolites detected in the flesh and nine in the liver, along with a number of ‘undescribed’ metabolites. The CTX1B levels in the flesh of all seven GME specimens were above the USFDA guidance limit of 0.01 µg/kg CTX1B equivalence [22]. There were 21 elements detected in the GME flesh samples, including those of volcanic nature, rubidium, and strontium. Of the heavy metals, there were elevated levels of total arsenic, while only trace levels of mercury. Nutritionally, the GMEs were shown to be a lean protein source; however, due to the bioaccumulation of CTXs, they pose a substantial food safety risk. Further research is now required to investigate the ‘unknown’ metabolites detected in the GME specimens and ascertain if they also pose a risk to human health.

## Figures and Tables

**Table 1:** Ciguatoxin levels (µg/kg) observed in the flesh and liver of the seven giant moray eel (*Gymnothorax javanicus*) specimens collected from Muri Lagoon (Rarotonga, Cook Islands). Age (years) was determined for the three largest specimens.

Giant Moray Eel														
CTX Analog	1		2		3		4		5		6		7	
	F	L	F	L	F	L	F	L	F	L	F	L	F	L
CTX1B	**0.04**	**1.9**	**0.07**	**3.1**	D	**2.4**	D	**2.1**	D	**1.5**	D	**0.50**	**0.04**	**3.9**
52-*epi*-54-deoxy CTX1B	D	0.50	D	0.36	D	0.43	D	0.35	D	0.28	–	0.08	D	0.39
54-deoxyCTX1B	–	0.29	D	0.30	D	0.29	D	0.18	–	0.19	–	0.07	D	0.28
CTX4A	–	–	–	–	–	–	–	–	–	–	–	–	–	–
CTX4B	–	–	–	–	–	–	–	–	–	–	–	–	–	–
M-*seco*-CTX4A/B	–	–	–	–	–	–	–	–	–	–	–	–	–	–
51-OH-CTX3C	–	D	–	0.25	–	0.32	–	0.20	–	0.17	–	–	–	0.07
2,3-diOH-CTX3B	0.19	31	0.34	54	0.12	20	0.05	16	D	14	D	6.0	0.15	28
2,3-diOH-CTX3C	0.14	34	0.22	55	0.06	18	D	18	D	12	D	8.4	0.04	29
CTX3B	0.13	1.4	0.53	3.2	0.15	2.59	0.12	2.7	0.11	2.3	0.04	0.38	0.27	2.6
CTX3C	0.05	0.71	0.24	1.6	0.07	1.31	0.05	1.3	0.04	0.95	D	0.16	0.13	0.88
M-*seco*-CTX3B/C	D	0.13	D	0.21	D	0.23	–	0.24	D	0.16	–	0.03	D	0.25
Total CTXs ^a^	0.58	70	1.5	118	0.44	46	0.27	41	0.22	32	0.10	16	0.65	66
GME age (years)	NC		39		26		NC		NC		NC		23	

F = flesh; L = liver; CTX = ciguatoxin; D = detected (above the limit of detection and below the limit of quantitation); – = not detected; OH = hydroxy; NC = not collected. Bolded values are above the United States Food and Drug Administration guidance level of 0.01 µg/kg CTX1B equiveillance [22]. There is no guidance level for the other CTX analogs. ^a^ Expressed as a sum of all CTXs quantified.

**Table 2:** Ciguatoxin analogs tentatively identified (based on published multiple reaction monitoring transitions and relative retention times; detected/not detected) in the flesh and liver of the seven giant moray eel (*Gymnothorax javanicus*) specimens collected from Muri Lagoon (Rarotonga, Cook Islands).

	Giant Moray Eel													
CTX Analog	1		2		3		4		5		6		7	
	F	L	F	L	F	L	F	L	F	L	F	L	F	L
M-*seco*-CTX3B/C methyl acetate	D	D	D	D	D	D	D	D	D	D	D	D	–	D
2-OH-CTX3C	–	D	–	–	–	D	–	D	–	–	–	D	–	D
51-OH-2-oxo-CTX3C	–	–	–	–	–	–	–	–	–	–	–	–	–	–
A-seco-51-OH-CTX3C	–	–	–	–	–	–	–	–	–	–	–	–	–	–
2,3,51-triOH-CTX3C	–	D	–	D	–	D	–	D	–	D	–	D	–	D
7-oxo-CTX1B	–	D	–	D	–	D	–	D	–	D	–	D	–	D
7-OH-CTX1B	–	D	–	D	–	D	–	D	–	D	–	D	–	D
4-OH-7-oxo-CTX1B	–	D	–	D	–	D	–	D	–	D	–	D	–	D

F = flesh; L = liver; CTX = ciguatoxin; D = detected (above the limit of detection and below the limit of quantitation); – = not detected; OH = hydroxy.

**Table 3:** Ciguatoxin fortification experiments of a solvent blank and the flesh and liver from giant moray eel (*Gymnothorax javanicus*) specimens collected from Muri Lagoon (Rarotonga, Cook Islands) to assess extraction recovery (full fortification), and to assess matrix suppression and enhancement effects (post-solid phase extraction fortification).

			Recovery (%)			
	Matrix	GME	CTX1B	CTX3B	CTX3C	CTX4A
Full fortification	Solvent	–	88	87	90	87
	Flesh	1	68	24	26	24
		4	54	19	24	28
	Liver	2	69	47	39	9
Post SPE fortification	Flesh	1	95	74	78	86
		5	90	88	89	86
		7	87	93	95	97
	Liver	6	44	94	79	91

GME = giant moray eel; CTX = ciguatoxin; SPE = solid phase extraction.

**Table 4:** Elemental composition (mg/kg) of the flesh from seven giant moray eel (*Gymnothorax javanicus*) specimens collected from Muri Lagoon (Rarotonga, Cook Islands).

Element ^a^	Giant Moray Eel						
	1	2	3	4	5	6	7
Arsenic	2.5	1.6	1.9	2.9	4.9	3.3	2.0
Barium	0.14	0.045	0.04	0.036	0.037	0.019	0.014
Caesium	0.037	0.026	0.025	0.027	0.029	0.024	0.026
Calcium	2000	800	1400	1600	1700	1600	680
Chromium	1	0.84	0.44	0.36	0.22	0.31	0.22
Copper	0.79	0.42	0.56	0.61	0.37	0.4	0.37
Iron	7.6	7.4	4.2	3.7	2.6	2.9	3.4
Lithium	0.028	0.015	0.024	0.023	0.025	0.023	0.013
Magnesium	260	230	210	240	250	240	220
Manganese	0.12	0.093	0.073	0.035	0.095	0.035	0.057
Mercury	0.036	0.082	0.082	0.046	0.073	0.066	0.12
Molybdenum	0.030	0.024	0.012	–	–	–	–
Nickel	0.036	0.033	–	–	–	0.026	0.023
Phosphorus	2900	2100	2200	2500	2600	2500	2000
Potassium	4400	3900	3600	4000	4000	3900	3900
Rubidium	0.78	0.67	0.66	0.76	0.75	0.69	0.64
Selenium	0.5	0.44	0.54	0.44	0.52	0.5	0.52
Sodium	730	750	640	770	780	800	630
Strontium	8.9	3.5	6.3	7	7.9	7.1	3
Sulphur	2500	2200	1700	2300	2100	2200	1900
Zinc	21	18	15	19	18	18	16

^a^ Additional elements analyzed but not detected (limit of detection [LoD] in brackets): Antimony (<0.01), Beryllium (<0.004), Bismuth (<0.004), Boron (<1), Cadmium (<0.009), Cobalt (<0.01), Lanthanum (<0.01), Lead (<0.01), Thallium (<0.004), Tin (<0.02), Uranium (<0.002), Vanadium (<0.04), and Yttrium (<0.004). – = Not detected (Molybdenum [<0.01] and Nickel [<0.02]).

**Table 5:** Proximate composition (g/100 g) showing the fat, protein, moisture, ash, and carbohydrate levels in the seven giant moray eel (*Gymnothorax javanicus*) specimens collected from Muri Lagoon (Rarotonga, Cook Islands).

Proximate	Giant Moray Eel						
	1	2	3	4	5	6	7
Fat	2.4	5.8	6.6	1.8	3.1	2.6	4.6
Protein	20.4	18.9	18.1	19.8	19.9	19.6	19.3
Moisture	76.7	75.6	74.5	77.3	75.7	77.5	75.0
Ash	1.5	1.2	1.7	1.3	1.1	1.5	1.3
Carbohydrate	<0.1	<0.1	<0.1	<0.1	<0.1	<0.1	<0.1

**Table 6:** Physical measurements and observations of the seven giant moray eel (*Gymnothorax javanicus*) specimens collected from Muri Lagoon (Rarotonga, Cook Islands).

	Giant Moray Eel						
	1	2	3	4	5	6	7
Total length (cm)	123	165	155	120	130	136	152
Width at widest point (cm)	14	24	23	13	19	14	22
Width at anus (cm)	12	18	19	11	14	13	19
Flesh weight (g)	2068	5330	4630	1912	3112	2441	5234
Liver length (cm)	24.5	35.5	34	24	27.5	26.5	33.5
Liver weight (g)	37	144	143	33	99	56	120
Sex (tentative assignment)	m	f	f	m	m	m	f
Age (years)	–	39	26	–	–	–	23

## Data Availability

The data presented in this study are included in this article/Supplementary Materials; further inquiries can be directed to the corresponding authors.

## Supplementary Materials

The following supporting information can be downloaded at https://www.mdpi.com/article/10.3390/md23090341/s1. Figure S1. X-rays of the GME 3 skull showing the (A) left lateral view—jaw closed, (B) right lateral jaw—jaw closed, (C) left lateral view—jaw fully extended, and (D) right lateral view—jaw fully extended; Figure S2. X-rays of the GME 7 skull showing the (A) left lateral view—jaw closed, (B) right lateral jaw—jaw closed, (C) left lateral view—jaw fully extended, and (D) right lateral view—jaw fully extended; Figure S3. The sagitta otoliths of GME 3 showing the (A) lefthand concave dorsal depression, (B) righthand concave dorsal depression, (C) lefthand sulcal groove, and (D) righthand sulcal groove; Figure S4. The sagitta otoliths of GME 7 showing the (A) lefthand concave dorsal depression, (B) righthand concave dorsal depression, (C) lefthand sulcal groove, and (D) righthand sulcal groove; Figure S5. A cross-section of the left-hand sagitta otolith used to age the GME 3 specimen; Figure S6. A cross-section of the left-hand sagitta otolith used to age the GME 7 specimen; Figure S7. The seven giant moray eel specimens used in this study; Table S1. The quantified levels of ciguatoxins (µg/kg) found in the localized bioaccumulation study with the giant moray eel specimens; Table S2. List of the Type I and Type II ciguatoxins monitored, including multiple reaction monitoring transitions, electrospray ionization mode, cone voltage, collision energy, and dwell times; Table S3. List of the gambierones, maitotoxins, gambieric acids, gambierol, and gambieroxide monitored, including multiple reaction monitoring transitions, electrospray ionization mode, cone voltage, collision energy, and dwell times; Table S4. List of the palytoxin-like compounds monitored for the intact method, including multiple reaction monitoring transitions, electrospray ionization mode, cone voltage, collision energy, and dwell times; Table S5. List of the palytoxin-like compounds monitored for the oxidative cleavage method, including multiple reaction monitoring transitions, electrospray ionization mode, cone voltage, collision energy, and dwell times; Table S6. List of the other toxins monitored, including multiple reaction monitoring transitions, electrospray ionization mode, cone voltage, collision energy, and dwell times; Table S7. List of the available certified standards, and associated relative response factors and toxicity equivalence factors, for the metabolites analyzed as part of the ‘other toxin classes’ method.
