# Supplementary material for: Giant Moray Eel (Gymnothorax javanicus), a Long-Living Apex Predator That Poses a Food Safety Risk in the Pacific
Source: Mar Drugs. 2025 Aug 26;23(9):341. doi: 10.3390/md23090341 (PMC12472033; doi:10.3390/md23090341)
Supplement: Supplementary file 1 [file marinedrugs-23-00341-s001.zip › marinedrugs-3813742-supplementary.pdf]

Supplementary information for

Giant moray eel (*Gymnothorax javanicus*), a long living apex predator that poses a food safety risk in the Pacific

Emillie M.F. Passfield,* Kirsty F. Smith, D. Tim Harwood, Joshua D. Fitzgerald, Phoebe A. Argyle, Jacob Thomson-Laing and J. Sam Murray*

* Corresponding authors –

emillie.passfield@cawthron.org.nz; sam.murray@cawthron.org.nz

Figure S1. X-rays of the GME 3 skull showing the A) left lateral view - jaw closed, B) right lateral jaw - jaw closed, C) left lateral view - jaw fully extended, and D) right lateral view - jaw fully extended. 4

Figure S2. X-rays of the GME 7 skull showing the A) left lateral view - jaw closed, B) right lateral jaw - jaw closed, C) left lateral view - jaw fully extended, and D) right lateral view - jaw fully extended. 4

Figure S3. The sagitta otoliths of GME 3 showing the A) lefthand concave dorsal depression, B) righthand concave dorsal depression, C) lefthand sulcal groove, and D) righthand sulcal groove. 5

Figure S4. The sagitta otoliths of GME 7 showing the A) lefthand concave dorsal depression, B) righthand concave dorsal depression, C) lefthand sulcal groove, and D) righthand sulcal groove. 6

Figure S5. A cross section of the lefthand sagitta otolith used to age the GME 3 specimen................................ 7

Figure S6. A cross section of the lefthand sagitta otolith used to age the GME 7 specimen................................ 8

Figure S7. The seven giant moray eel specimens used in this study. 9

Table S1. The quantified levels of ciguatoxins (µg/kg) found in the localized bioaccumulation study with the giant moray eel specimens 10

Table S2. List of the Type I and Type II ciguatoxins monitored, including multiple reaction monitoring transitions, electrospray ionization mode, cone voltage, collision energy and dwell times 11

Table S3 List of the gambierones, maitotoxins, gambieric acids, gambierol and gambieroxide monitored, including multiple reaction monitoring transitions, electrospray ionization mode, cone voltage, collision energy and dwell times 12

Table S4. List of the palytoxin-like compounds monitored for the intact method, including multiple reaction monitoring transitions, electrospray ionization mode, cone voltage, collision energy and dwell times........ 13

Table S5. List of the palytoxin-like compounds monitored for the oxidative cleavage method, including multiple reaction monitoring transitions, electrospray ionization mode, cone voltage, collision energy and dwell times 14

Table S6. List of the other toxins monitored, including multiple reaction monitoring transitions, electrospray ionization mode, cone voltage, collision energy and dwell times 15

Table S7. List of the available certified standards, and associated relative response factors and toxicity equivalence factors, for the metabolites analysed as part of the 'other toxin classes' method 16

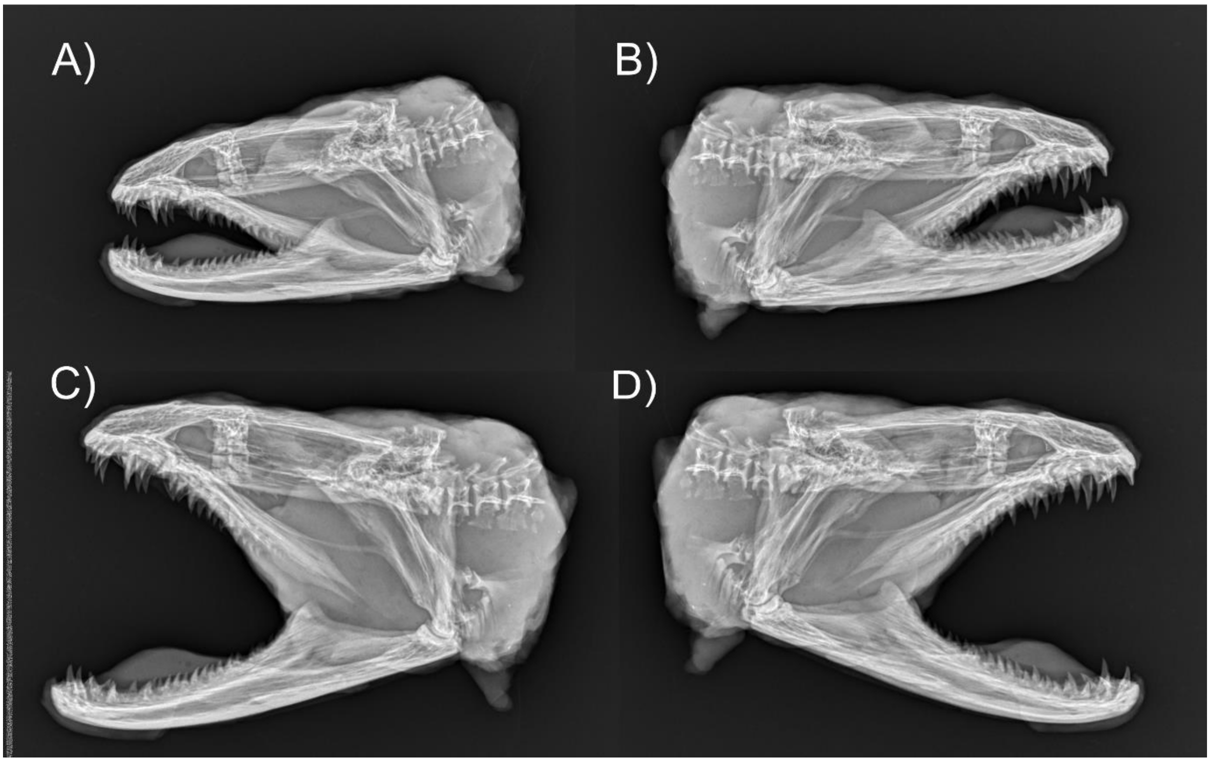

Figure S1. X-rays of the GME 3 skull showing the A) left lateral view - jaw closed, B) right lateral jaw - jaw closed, C) left lateral view - jaw fully extended, and D) right lateral view - jaw fully extended.

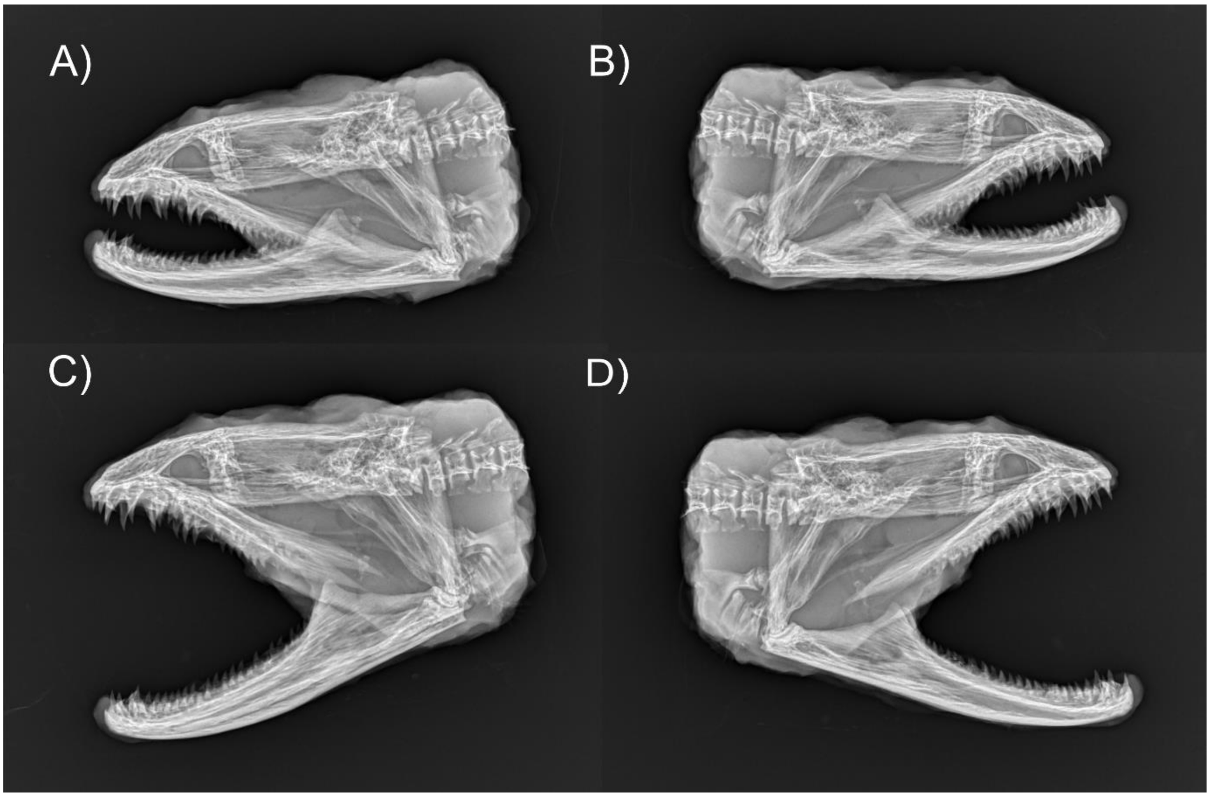

Figure S2. X-rays of the GME 7 skull showing the A) left lateral view - jaw closed, B) right lateral jaw - jaw closed, C) left lateral view - jaw fully extended, and D) right lateral view - jaw fully extended.

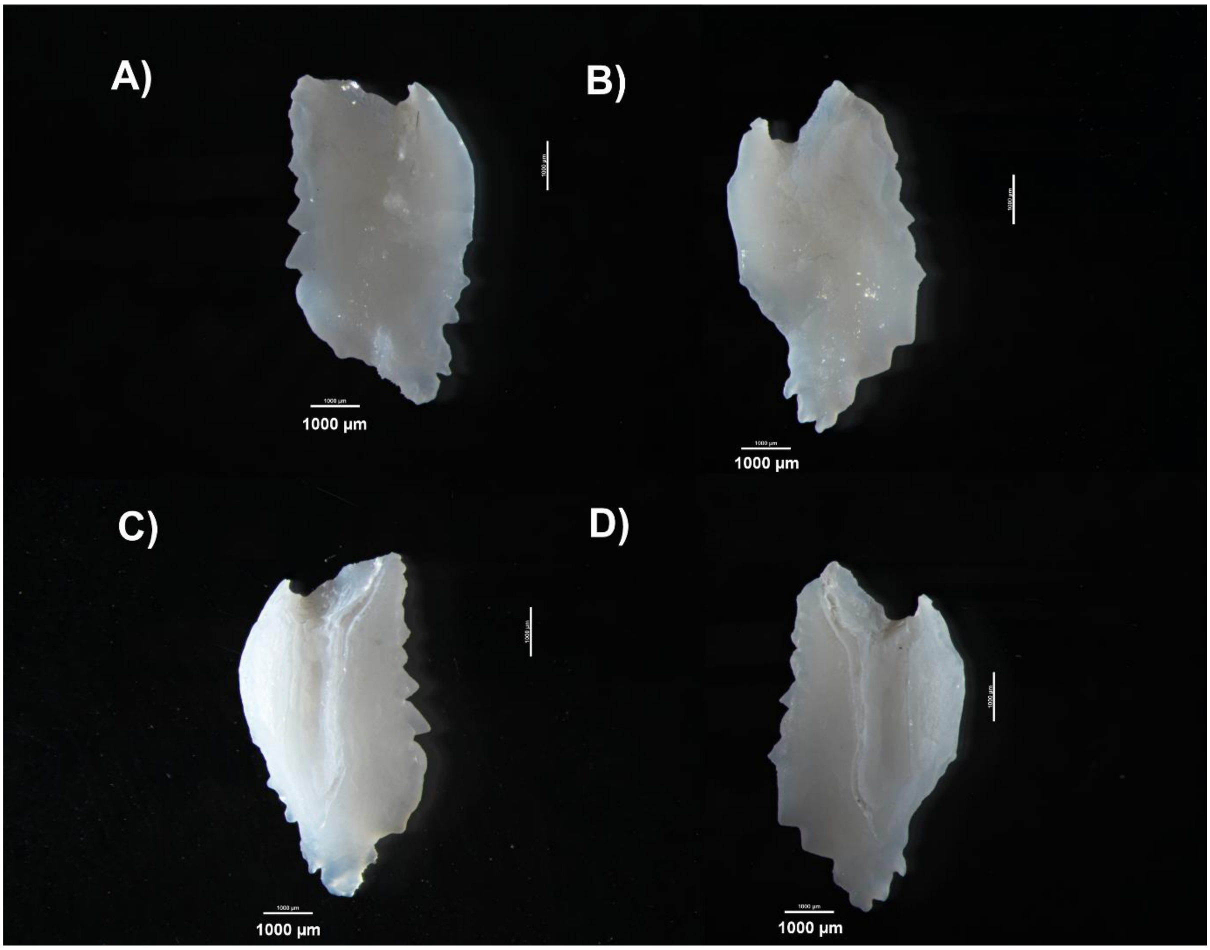

Figure S3. The sagitta otoliths of GME 3 showing the A) lefthand concave dorsal depression, B) righthand concave dorsal depression, C) lefthand sulcal groove, and D) righthand sulcal groove.

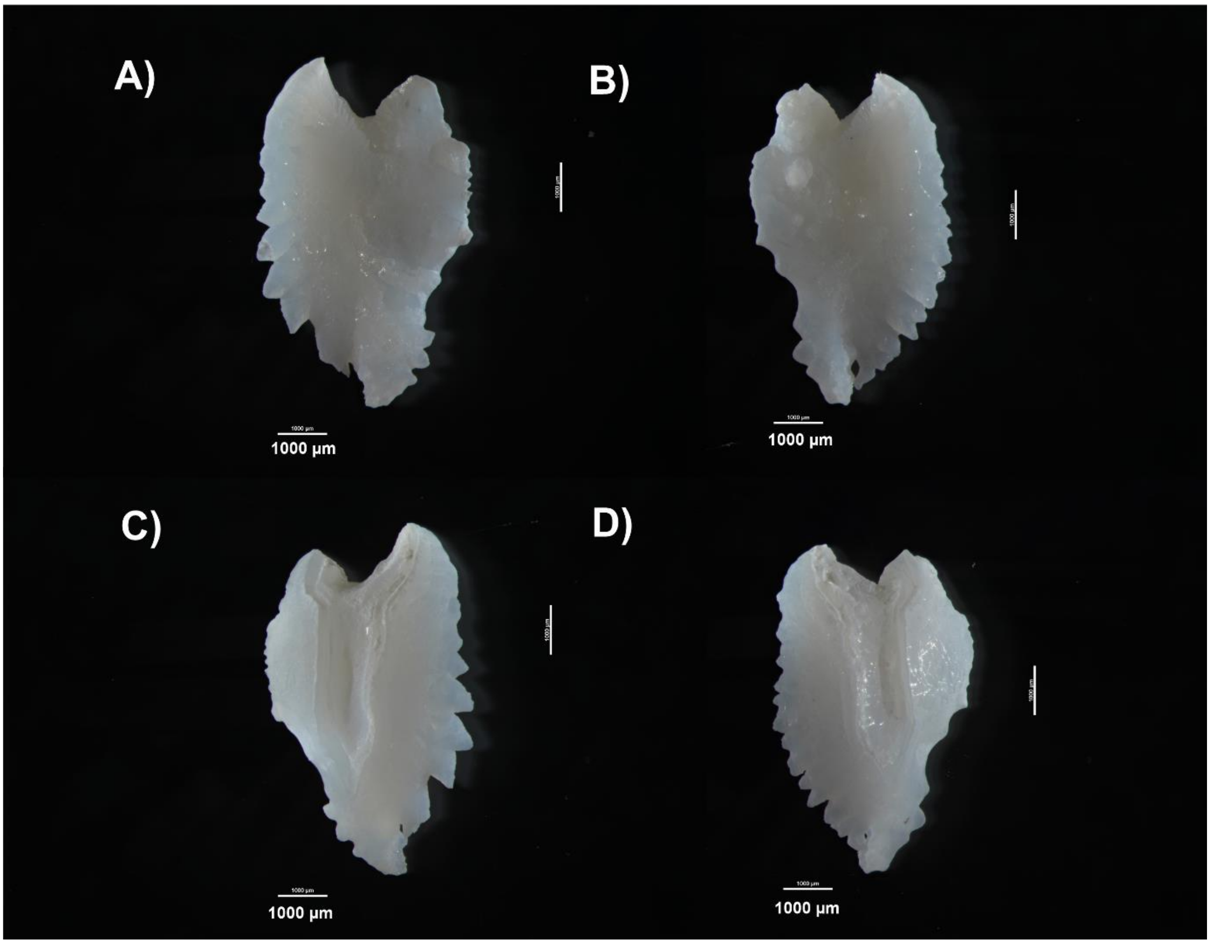

Figure S4. The sagitta otoliths of GME 7 showing the A) lefthand concave dorsal depression, B) righthand concave dorsal depression, C) lefthand sulcal groove, and D) righthand sulcal groove.

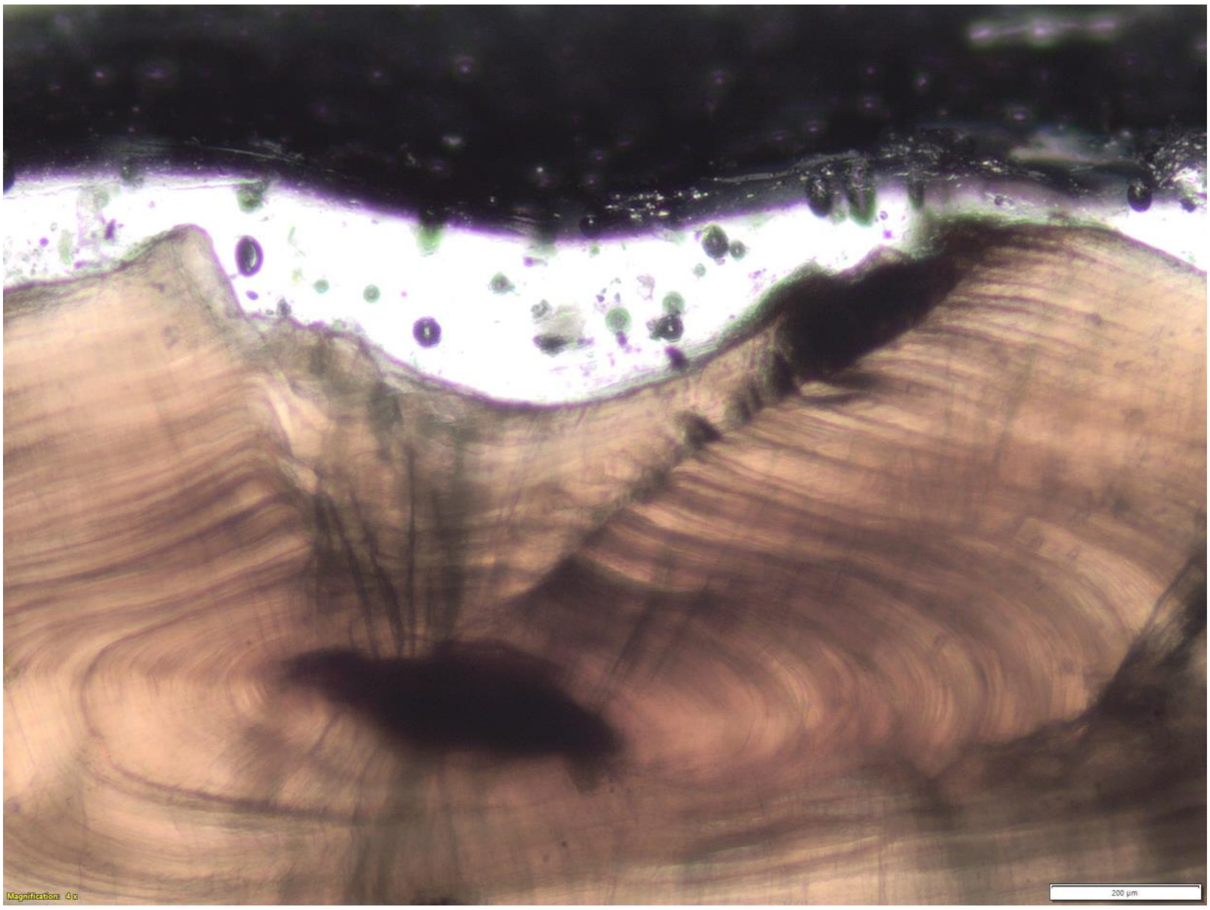

Figure S5. A cross section of the lefthand sagitta otolith used to age the GME 3 specimen.

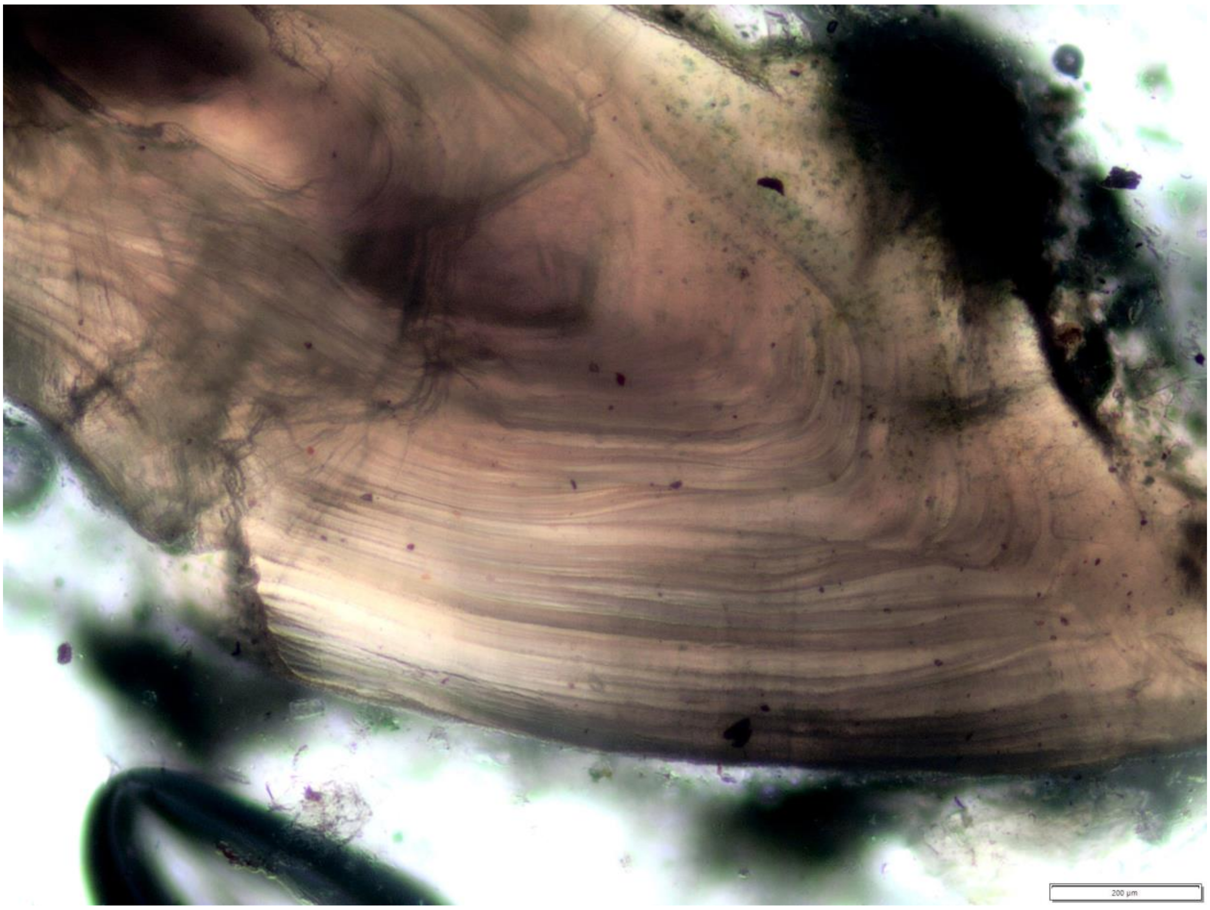

Figure S6. A cross section of the lefthand sagitta otolith used to age the GME 7 specimen.

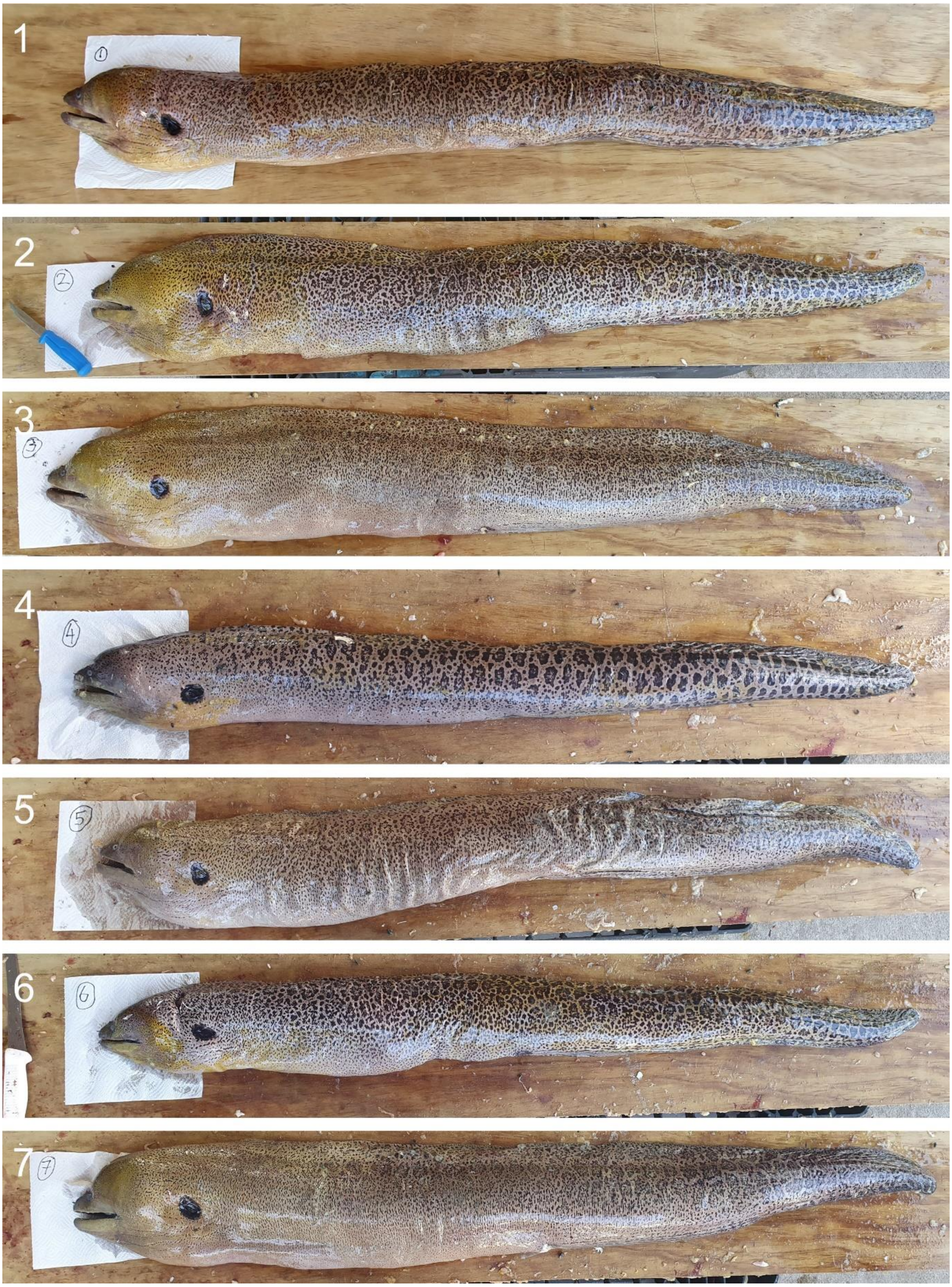

Figure S7. The seven giant moray eel specimens used in this study

Table S1. The quantified levels of ciguatoxins (µg/kg) found in the localized bioaccumulation study with the giant moray eel specimens

Eel	Portion	CTX1B	52-*epi*-54-deoxyCTX1B	54-deoxyCTX1B	51-hydroxy-CTX3C	2,3-dihydroxy-CTX3B	2,3-dihydroxy-CTX3C	CTX3B	CTX3C	M-seco-CTX3B/C	Total CTXs
GME 1	A	0.070	D			0.19	0.054	0.17	0.052	D	0.54
	B	0.047	D			0.14	0.039	0.11	0.032	D	0.37
	C	0.053	D			0.16	0.050	0.11	0.026	D	0.40
	D	0.061	D			0.14	0.060	0.096	0.035	D	0.40
GME 2	A	0.10	D			0.42	0.29	0.34	0.098	D	1.24
	B	0.12	D	D	0.049	0.56	0.30	0.38	0.11	D	1.52
	C	0.10	D	D	0.060	0.44	0.16	0.25	0.089	D	1.11
	D	0.090	D			0.45	0.19	0.34	0.088	D	1.16
	Head	0.11	D		0.061	0.36	0.16	0.37	0.10	D	1.17
GME 3	A	0.048	D	D		0.24	0.096	0.14	0.049	D	0.57
	B	0.038	D			0.16	0.077	0.13	0.049	D	0.45
	C	0.046	D			0.19	0.054	0.090	0.040	D	0.42
	D	D	D			0.12	0.066	0.078	0.042	D	0.31
	Head	D	D		D	0.11	0.044	0.091	0.028	D	0.27
GME 4	A	0.041	D			0.060	0.028	0.12	0.039	D	0.28
	B	0.034	D			0.089		0.076	0.029	D	0.23
	C	D				0.033		0.043	0.021	D	0.10
	D	D	D			0.047		0.10	0.029	D	0.18
GME 5	A	D				0.074		0.050	D	D	0.12
	B	D	D			0.088	D	0.074	0.047	D	0.21
	C	D				0.047	D	0.049	D	D	0.10
	D	D				0.072	D	0.044	D	D	0.12
GME 6	A	D				0.075	0.040				0.11
	B	D				0.055		D	D		0.06
	C	D				0.075	0.040	D			0.11
	D	D				0.039		D			0.04
GME 7	A	0.059	D	D		0.16	0.053	0.084	0.027	D	0.38
	B	0.054	D			0.19	0.044	0.093	0.030	D	0.41
	C	0.046	D			0.11	0.048	0.060	0.023	D	0.29
	D	0.057	D			0.17	0.037	0.11	0.041	D	0.41
	Head	0.043	D			0.14	0.054	0.098	0.032		0.37

GME = Giant moray eel; CTX = ciguatoxin; D = detected; colour scale from lowest CTX content (green) to highest (red); results rounded to two significant figures.

Table S2. List of the Type I and Type II ciguatoxins monitored, including multiple reaction monitoring transitions, electrospray ionization mode, cone voltage, collision energy and dwell times

**Analogue**	**Acquisition time (min)**	**MRM**	**ESI Mode**	**CE (eV)**	**Cone (V)**	**Dwell (secs)**
CTX4A/B and M-seco ($[M+H–H_2O]^+$)	3.5 - 8	1,061.6>125.1	+ve	50	30	0.013
CTX4A/B and M-seco ($[M+H–H_2O]^+$)	*3.5 - 8*	*1,061.6>155.1*	*+ve*	*50*	*30*	*0.013*
CTX1B	3.2 – 5.0	1,128.6>95.1	+ve	65	30	0.013
CTX1B	*3.2 – 5.0*	*1,128.6>109*	*+ve*	*55*	*30*	*0.013*
CTX1B	*3.2 – 5.0*	*1,133.6>1,133.6*	*+ve*	*55*	*75*	*0.013*
52-*epi*-54-deoxyCTX1B	4.1 – 6.2	1,112.6>1,041.8	+ve	25	30	0.013
52-epi-54-deoxyCTX1B	*4.1 – 6.2*	*1,112.6>1,077.8*	*+ve*	*20*	*30*	*0.013*
52-epi-54-deoxyCTX1B	*4.1 – 6.2*	*1,117.6>1,117.6*	*+ve*	*55*	*75*	*0.013*
54-deoxyCTX1B	4.1 – 6.2	1,112.6>1,041.8	+ve	25	30	0.013
54-deoxyCTX1B	*4.1 – 6.2*	*1,112.6>1,077.8*	*+ve*	*20*	*30*	*0.013*
54-deoxyCTX1B	*4.1 – 6.2*	*1,117.6>1,117.6*	*+ve*	*55*	*75*	*0.013*
7-oxo-CTX1B	1 – 8.5	1,144.6>95.1	+ve	65	30	0.019
7-oxo-CTX1B	*1 – 8.5*	*1,144.6>109*	*+ve*	*50*	*30*	*0.019*
7-oxo-CTX1B	*1 – 8.5*	*1,149.6>1,149.6*	*+ve*	*50*	*75*	*0.019*
7-hydroxy-CTX1B	1 – 8.5	1,146.6>95.1	+ve	65	30	0.019
7-hydroxy-CTX1B	*1 – 8.5*	*1,146.6>109*	*+ve*	*50*	*30*	*0.019*
7-hydroxy-CTX1B	*1 – 8.5*	*1,151.6>1,151.6*	*+ve*	*50*	*75*	*0.019*
4-hydroxy-7-oxo-CTX1B	1 – 8.5	1,162.6>95.1	+ve	65	30	0.019
4-hydroxy-7-oxo-CTX1B	*1 – 8.5*	*1,162.6>109*	*+ve*	*50*	*30*	*0.019*
4-hydroxy-7-oxo-CTX1B	*1 – 8.5*	*1,167.6>1,167.6*	*+ve*	*50*	*75*	*0.019*
CTX3B/C and M-seco ($[M+H–H_2O]^+$)	3.5 - 8	1,023.6>125.1	+ve	50	30	0.013
CTX3B/C and M-seco ($[M+H–H_2O]^+$)	*3.5 - 8*	*1,023.6>155.1*	*+ve*	*50*	*30*	*0.013*
M-seco-CTX3C methyl acetate	1 – 8.5	1,055.6>157.1	+ve	50	30	0.011
M-seco-CTX3C methyl acetate	*1 – 8.5*	*1,055.6>187.1*	*+ve*	*50*	*30*	*0.011*
51-hydroxy-CTX3C	4.3 - 6	1,039.6>141.1	+ve	50	30	0.013
51-hydroxy-CTX3C	*4.3 - 6*	*1,039.6>171.1*	*+ve*	*50*	*30*	*0.013*
51-hydroxy-CTX3C	*4.3 - 6*	*1,061.6>1,061.6*	*+ve*	*50*	*75*	*0.013*
2-hydroxy-CTX3C	4.35 - 6	1,039.6>125.1	+ve	50	30	0.013
2-hydroxy-CTX3C	*4.35 - 6*	*1,039.5>155.1*	*+ve*	*50*	*30*	*0.013*
2-hydroxy-CTX3C	*4.35 - 6*	*1,061.6>1,061.6*	*+ve*	*50*	*75*	*0.013*
51-hydroxy-2-oxo-CTX3C	1 – 8.5	1,055.6>141.1	+ve	50	30	0.011
51-hydroxy-2-oxo-CTX3C	*1 – 8.5*	*1,055.6>171.1*	*+ve*	*50*	*30*	*0.011*
51-hydroxy-2-oxo-CTX3C	*1 – 8.5*	*1,077.6>1,077.6*	*+ve*	*50*	*75*	*0.011*
2,3-dihydroxy-CTX3B/C	1 – 8.5	1,057.6>125.1	+ve	50	30	0.011
2,3-dihydroxy-CTX3B/C	*1 – 8.5*	*1,057.6>155.1*	*+ve*	*50*	*30*	*0.011*
2,3-dihydroxy-CTX3B/C	*1 – 8.5*	*1,079.6>1,079.6*	*+ve*	*50*	*75*	*0.011*
2,3,51-trihydroxy-CTX3C	1 – 8.5	1,073.6>141.1	+ve	50	30	0.011
2,3,51-trihydroxy-CTX3C	*1 – 8.5*	*1,073.6>171.1*	*+ve*	*50*	*30*	*0.011*
2,3,51-trihydroxy-CTX3C	*1 – 8.5*	*1,095.6>1,095.6*	*+ve*	*50*	*75*	*0.011*
A-seco-51-hydroxy-CTX3C	1 – 8.5	1,093.6>141.1	+ve	50	30	0.011
A-seco-51-hydroxy-CTX3C	*1 – 8.5*	*1,093.6>171.1*	*+ve*	*50*	*30*	*0.011*
A-seco-51-hydroxy-CTX3C	*1 – 8.5*	*1,115.6>1,115.6*	*+ve*	*50*	*75*	*0.011*

MRM = Multiple reaction monitoring; ESI = electrospray ionization; CE = collision energy; 'regular text' = quantitation channel; '*Italics text*' = confirmation channels; CTX = ciguatoxin.

Table S3. List of the gambierones, maitotoxins, gambieric acids, gambierol and gambieroxide monitored, including multiple reaction monitoring transitions, electrospray ionization mode, cone voltage, collision energy and dwell times

**Compound**	**Acquisition time (min)**	**MRM**	**ESI mode**	**CE (eV)**	**Cone (V)**	**Dwell (secs)**
Gambierone	1 - 4.0	1,023.3>96.8	-ve	50	40	0.019
Gambierone	*1 - 4.0*	*899.2>96.8*	*-ve*	*50*	*40*	*0.019*
Anhydrogambierone	1 - 4.0	1,005.3>96.8	-ve	50	40	0.019
Anhydrogambierone	*1 - 4.0*	*881.3>96.8*	*-ve*	*50*	*40*	*0.019*
Dianhydrogambierone	1 - 4.0	987.3>96.8	-ve	50	40	0.019
Dianhydrogambierone	*1 - 4.0*	*863.2>96.8*	*-ve*	*50*	*40*	*0.019*
Sulfo-gambierone	1 - 4.0	1,103.5>96.8	-ve	50	40	0.019
Dihydrosulfo-gambierone	1 - 4.0	1,105.5>96.8	-ve	50	40	0.019
44- and 29-MG	1 - 4.0	1,037.3>96.8	-ve	70	40	0.019
44- and 29-MG	*1 - 4.0*	*899.2>96.8*	*-ve*	*50*	*40*	*0.019*
38-deoxy-44-MG	1 - 4.0	1,019.3>96.8	-ve	70	40	0.019
38-deoxy-44-MG	*1 - 4.0*	*881.3>96.8*	*-ve*	*50*	*40*	*0.019*
Dianhydro-44-MG	1 - 4.0	1,001.3>96.8	-ve	70	40	0.019
Dianhydro-44-MG	*1 - 4.0*	*863.2>96.8*	*-ve*	*50*	*40*	*0.019*
12,13-dihydro-44-MG	1 - 4.0	1,039.5>96.8	-ve	70	40	0.019
38-deoxy-12,13-dihydro-44-MG	1 - 4.0	1,021.5>96.8	-ve	70	40	0.019
MTX-1	1 - 4.5	1,689.4>1,689.4	-ve	80	40	0.014
MTX-1	*1 - 4.5*	*1,126.1>96.8*	*-ve*	*100*	*40*	*0.014*
MTX-2	1 - 4.5	1,637.5>1,637.5	-ve	60	40	0.014
MTX-2	*1 - 4.5*	*1,098.6>96.8*	*-ve*	*100*	*40*	*0.014*
MTX-4	1 - 4.5	1,645.2>1,645.2	-ve	60	40	0.014
MTX-4	*1 - 4.5*	*1,096.5>96.8*	*-ve*	*100*	*40*	*0.014*
MTX-5	1 - 4.5	1,668.8>>1,668.8	-ve	40	40	0.014
MTX-5	*1 - 4.5*	*1,668.8>96.8*	*-ve*	*100*	*40*	*0.014*
MTX-6	1 - 4.5	1,656.3>1,656.3	-ve	60	40	0.014
MTX-6	*1 - 4.5*	*1,104.1>96.8*	*-ve*	*100*	*40*	*0.014*
MTX-7	1 - 4.5	1,671.4>1,671.4	-ve	60	40	0.014
MTX-7	*1 - 4.5*	*1,114.1>96.8*	*-ve*	*100*	*40*	*0.014*
Gambieric acid A	1 - 5	1,055.6>1,055.6	-ve	20	40	0.03
Gambieric acid A	*1 – 5*	*1,057.6>1,057.6*	*+ve*	*5*	*30*	*0.03*
Gambieric acid B	1 – 5	1,069.7>1,069.7	-ve	20	40	0.03
Gambieric acid B	*1 – 5*	*1,071.7>1,071.7*	*+ve*	*5*	*30*	*0.03*
Gambieric acid C	1 – 5	1,183.7>1,183.7	-ve	20	40	0.03
Gambieric acid C	*1 – 5*	*1,185.7>1,039.6*	*+ve*	*17*	*30*	*0.03*
Gambieric acid D	1 – 5	1,197.7>1,197.7	-ve	20	40	0.03
Gambieric acid D	*1 – 5*	*1,199.7>1,053.6*	*+ve*	*17*	*30*	*0.03*
Gambierol	1 – 5	755.5>755.5	-ve	20	40	0.03
Gambierol	*1 - 5*	*757.5>757.5*	*+ve*	*5*	*30*	*0.03*
Gambieroxide	1 – 4.5	1,193.6>96.8	-ve	60	40	0.03

MRM = Multiple reaction monitoring; ESI = electrospray ionization; CE = collision energy; 'regular text' = quantitation channel; *Italics text'* = confirmation channels; 44-MG = 44-methylgambierone, MTX = maitotoxin.

Table S4. List of the palytoxin-like compounds monitored for the intact method, including multiple reaction monitoring transitions, electrospray ionization mode, cone voltage, collision energy and dwell times

Compound	Acquisition time (min)	MRM	ESI mode	CE (eV)	Cone (V)	Dwell (secs)
OST-d	1.5 - 5	861.4 > 313.2	+ve	20	30	0.015
OST-d	*1.5 - 5*	*861.4 > 76.1*	*+ve*	*40*	*30*	*0.015*
OST-d	*1.5 - 5*	*1316.7 > 185.1*	*-ve*	*70*	*30*	*0.015*
OVTX-a	1.5 - 5	865.5 > 327.2	+ve	20	30	0.015
OVTX-a	*1.5 - 5*	*865.5 > 76.1*	*+ve*	*40*	*30*	*0.015*
OVTX-a	*1.5 - 5*	*1322.7 > 199.1*	*-ve*	*70*	30	*0.015*
OVTX-b	1.5 - 5	880.1 > 371.2	+ve	20	30	0.015
OVTX-b	*1.5 - 5*	*880.1 > 119.5*	*+ve*	*40*	*30*	*0.015*
OVTX-b	*1.5 - 5*	*1344.8 > 199.1*	*-ve*	*70*	*30*	*0.015*
OVTX-c	1.5 - 5	885.5 > 371.2	+ve	20	30	0.015
OVTX-c	*1.5 - 5*	*885.5 > 119.5*	*+ve*	*40*	*30*	*0.015*
OVTX-c	*1.5 - 5*	*1352.7 > 199.1*	*-ve*	*70*	*30*	*0.015*
OVTX-d, deoxyPLTX	1.5 - 5	870.8 > 327.2	+ve	20	30	0.015
OVTX-e	1.5 - 5	870.8 > 343.2	+ve	20	30	0.015
OVTX-d & e, deoxyPLTX	*1.5 - 5*	*870.8 > 76.1*	*+ve*	*40*	*30*	*0.015*
OVTX-d & e, deoxyPLTX	*1.5 - 5*	*1330.7 > 199.1*	*-ve*	*70*	30	*0.015*
OVTX-f	1.5 - 5	874.8 > 327.2	+ve	20	30	0.015
OVTX-f	*1.5 - 5*	*874.8 > 76.1*	*+ve*	*40*	*30*	*0.015*
OVTX-f	*1.5 - 5*	*1336.8 > 199.1*	*-ve*	*70*	*30*	*0.015*
PLTX	1.5 - 5	876.1 > 327.2	+ve	20	*30*	0.015
PLTX	*1.5 - 5*	*876.1 > 76.1*	*+ve*	*40*	*30*	*0.015*
PLTX	*1.5 - 5*	*1338.7 > 199.1*	*-ve*	*70*	*30*	*0.015*
42-hydroxy PLTX	1.5 - 5	881.5 > 327.2	+ve	20	30	0.015
42-hydroxy PLTX	*1.5 - 5*	*881.5 > 76.1*	*+ve*	*40*	*30*	*0.015*
42-hydroxy PLTX	*1.5 - 5*	*1346.7 > 199.1*	*-ve*	*70*	*30*	*0.015*

MRM = Multiple reaction monitoring; ESI = electrospray ionization; CE = collision energy; 'regular text' = quantitation channel; '*Italics text*' = confirmation channels; OST = ostreocin; OVTX = ovatoxin; PLTX = palytoxin.

Table S5. List of the palytoxin-like compounds monitored for the oxidative cleavage method, including multiple reaction monitoring transitions, electrospray ionization mode, cone voltage, collision energy and dwell times

**Compound**	**Acquisition time (min)**	**MRM**	**ESI Mode**	**CE (eV)**	**Cone (V)**	**Dwell (secs)**
Common amine fragment	0 - 4	300.2 > 107.0	+ve	25	30	0.05
OSTs	0 - 4	329.2 > 76.0	+ve	13	30	0.05
PLTX, OVTX-a, -d, -f & -g	0 - 4	343.2 > 76.0	+ve	13	30	0.05
OVTX-b & -c	0 - 4	387.2 > 119.5	+ve	13	30	0.05
OVTX-e, isobaricPLTX	0 - 4	327.2 > 60.0	+ve	13	30	0.05
HomoPLTX	0 - 4	357.2 > 90.0	+ve	13	30	0.05
BishomoPLTX	0 - 4	371.2 > 104.0	+ve	13	30	0.05

MRM = Multiple reaction monitoring channel used for quantitation; ESI = electrospray ionization; CE = collision energy; OST = ostreocin; OVTX = ovatoxin; PLTX = palytoxin.

Table S6. List of the other toxins monitored, including multiple reaction monitoring transitions, electrospray ionization mode, cone voltage, collision energy and dwell times

**Compound**	**Acquisition time (min)**	**MRM**	**ESI Mode**	**CE (eV)**	**Cone (V)**	**Dwell (secs)**
Domoic acid	0.80-1.10	312.10>266.10	+ve	16	30	0.100
Domoic acid	*0.80-1.10*	*312.10>161.00*	+ve	*25*	*30*	*0.100*
Gymnodimine	1.10-2.00	508.40>490.30	+ve	25	40	0.040
Spirolide Desmethyl C	1.10-2.00	692.50>164.10	+ve	50	50	0.040
Spirolide Desmethyl D	1.10-2.00	694.50>164.10	+ve	50	50	0.040
Pinnatoxin A	1.10-2.00	712.50>164.10	+ve	50	50	0.040
Pinnatoxin D	1.10-2.00	782.50>164.10	+ve	50	50	0.040
Pinnatoxin E	1.10-2.00	784.50>164.10	+ve	50	50	0.040
Pinnatoxin F	1.10-2.00	766.50>164.10	+ve	50	50	0.040
Pinnatoxin G	1.10-2.00	694.50>164.10	+ve	50	50	0.040
Brevetoxin B2	2.00-2.60	1034.60>929.50	+ve	35	60	0.150
Desoxy Brevetoxin B2	2.00-2.60	1018.60>204.10	+ve	45	60	0.150
Azaspiracid-1	2.60-4.50	842.50>654.50	+ve	55	50	0.012
Azaspiracid-2	2.60-4.50	856.50>645.50	+ve	45	50	0.012
Azaspiracid-3	2.60-4.50	828.50>640.50	+ve	55	50	0.012
Pectenotoxin-2	2.60-4.50	876.60 >823.50	+ve	25	40	0.012
Pectenotoxin-1	2.60-4.50	892.50>839.50	+ve	25	40	0.012
Pectenotoxin-11	2.60-4.50	892.50>839.50	+ve	25	40	0.012
Pectenotoxin-6	2.60-4.50	906.50>853.50	+ve	25	40	0.012
Pectenotoxin-2 seco acid a	2.60-4.50	894.50>823.50	+ve	25	40	0.012
Pectenotoxin-2 seco acid b	2.60-4.50	894.50>823.50	+ve	25	40	0.012
Free Okadaic acid	2.60-4.50	827.50>723.40	+ve	50	70	0.012
Free Okadaic acid	2.60-4.50	*803.50>255.20*	*-ve*	*45*	*80*	*0.020*
Free Dinophysistoxin-2	2.60-4.50	827.50>723.40	+ve	50	70	0.012
Free Dinophysistoxin-2	2.60-4.50	*803.50>255.20*	*-ve*	*45*	*80*	*0.020*
Free Dinophysistoxin-1	2.60-4.50	841.50>737.40	+ve	50	70	0.012
Free Dinophysistoxin-1	2.60-4.50	*817.50>255.20*	*-ve*	*45*	*80*	*0.020*
**Total Okadaic acid**	**0.80-2.00**	**803.50>255.20**	-ve	**45**	**80**	**0.029**
Total Okadaic acid	***0.80-2.00***	***827.50>723.40***	***+ve***	***50***	***70***	***0.029***
**Total Dinophysistoxin-2**	**0.80-2.00**	**803.50>255.20**	-ve	**45**	**80**	**0.029**
Total Dinophysistoxin-2	***0.80-2.00***	***827.50>723.40***	***+ve***	***50***	***70***	***0.029***
**Total Dinophysistoxin-1**	**0.80-2.00**	**817.50>255.20**	-ve	**45**	**80**	**0.029**
Total Dinophysistoxin-1	***0.80-2.00***	***841.50>737.40***	***+ve***	***50***	***70***	***0.029***
Yessotoxin	4.50-5.9	1141.50>1061.50	-ve	33	50	0.025
45-hydroxy-yessotoxin	2.60-4.50	1157.50>1077.50	*-ve*	33	50	0.012
Homo Yessotoxin	4.50-5.90	1155.50>1075.50	*-ve*	33	50	0.025
45-hydroxy-homo-yessotoxin	2.60-4.50	1171.50>1091.50	*-ve*	33	50	0.012

MRM = Multiple reaction monitoring; ESI = electrospray ionization; CE = collision energy; 'regular text' = quantitation channel; '*Italics text*' = confirmation channels; 'bold text' = hydrolyzed extracts.

Table S7. List of the available certified standards, and associated relative response factors and toxicity equivalence factors, for the metabolites analysed as part of the 'other toxin classes' method

**Toxin**	**Standard (Y/N)**	**Calibration reference**	**RRF**	**TEF**
Domoic acid	Y	DA	1	1
Gymnodimine	Y	GYM	1	1
Spirolide Desmethyl C	Y	SPX-C	1	1
Spirolide Desmethyl D	N	SPX-C	*1*	*1*
Pinnatoxin A	N	SPX-C	*1*	*1*
Pinnatoxin D	N	SPX-C	*1*	*1*
Pinnatoxin E	N	SPX-C	*1*	*1*
Pinnatoxin F	N	SPX-C	*1*	*1*
Pinnatoxin G	N	SPX-C	*1*	*1*
Brevetoxin B2	N	dBTX-B2	1	1
Desoxy Brevetoxin B2	Y	dBTX-B2	1	1
Azaspiracid-1	N	SPX-C	7.93	1
Azaspiracid-2	N	SPX-C	8.58	0.7 (AZA-1 eq.)
Azaspiracid-3	N	SPX-C	7.98	0.5 (AZA-1 eq.)
Pectenotoxin-2	Y	PTX-2	1	1
Pectenotoxin-1	N	PTX-2	*1*	*1*
Pectenotoxin-11	N	PTX-2	*1*	*1*
Pectenotoxin-6	N	PTX-2	*1*	*1*
Pectenotoxin-2 seco acid a	N	PTX-2	*1*	*1*
Pectenotoxin-2 seco acid b	N	PTX-2	*1*	*1*
Okadaic acid	Y	OA	1	1
Dinophysistoxin-2	N	OA	1.02	0.5
Dinophysistoxin-1	N	OA	0.94	1
Total Okadaic acid	Y	OA	1	1
Total Dinophysistoxin-2	N	OA	0.91	0.5
Total Dinophysistoxin-1	N	OA	0.91	1
Yessotoxin	Y	YTX	1	1
45-hydroxy-yessotoxin	N	YTX	1	0.5
Homo yessotoxin	N	YTX	1.04	1
45-hydroxy-homo-yessotoxin	N	YTX	1	*0.5*

RRF = Relative response factor; TEF = toxicity equivalency factor; *Italics* = assumed value.
